# Blunted reward-related striatal activity and behavioral disinhibition as a pathway to adolescent cannabis and e-cigarette use

**DOI:** 10.3389/fradm.2026.1741884

**Published:** 2026-02-04

**Authors:** Patricio M. Viera Perez, Lauren D. Hill, Katharine E. Crooks, Benjelene D. Sutherland, Angela R. Laird, Elisa M. Trucco, Matthew T. Sutherland

**Affiliations:** 1 Department of Psychology, Florida International University, Miami, FL, United States,; 2 The Center for Children and Families, Florida International University, Miami, FL, United States,; 3 Department of Psychiatry and Behavioral Sciences, Vanderbilt University Medical Center, Nashville, TN, United States,; 4 Department of Physics, Florida International University, Miami, FL, United States,; 5 Department of Psychiatry, University of Michigan, Ann Arbor, MI, United States

**Keywords:** adolescence, alcohol, cannabis, e-cigarettes, externalizing, impulsivity, striatum, substance use

## Abstract

**Introduction:**

Adolescence is a period notable for increased risk-taking behaviors, including substance use (SU). Longitudinal work has linked behavioral disinhibition, particularly impulsive dispositions and externalizing tendencies with SU, but the underlying neurobiological manifestations remain less well-defined. This study examined whether individual differences in reward-related striatal activity and impulsivity predicted mental health (externalizing symptoms) and SU outcomes (cannabis, nicotine, alcohol) over a year later.

**Methods:**

Adolescents (*n* = 140; *M*_*age*_ = 14.9 years) from a larger longitudinal cohort completed a Monetary Incentive Delay (MID) fMRI task at baseline along with a measure of self-reported impulsivity. At follow-up, they reported externalizing symptoms and days of cannabis, e-cigarette, and alcohol use. Task behavior [response times [RTs], hit rates [HRs]] and striatal responses to anticipatory gain cues were extracted. Serial mediation models tested whether impulsivity and externalizing mediated an association between striatal activity and subsequent SU.

**Results:**

Behaviorally, gain cues elicited faster target-related RTs and higher HRs (vs. loss or neutral trials), and performance scaled with incentive magnitude. Gain (vs. neutral) cues elicited greater bilateral caudate activity where *more* left caudate activity correlated with faster RTs and lower impulsivity. Serial mediation revealed that *less* left striatal activity during reward anticipation linked with higher impulsivity, which predicted more subsequent externalizing symptoms that, in turn, linked with more cannabis [indirect effect = −0.01, 95%CI (−0.04, −0.001)] and e-cigarette use days [indirect effect = −0.02, 95% CI (–0.05, −0.004)]. No indirect or direct effects emerged for alcohol use.

**Conclusions:**

These findings suggest blunted striatal activity may reflect reduced motivational drive for lower-intensity rewards (e.g., fictitious MID monetary gains), which contribute to SU vulnerability via heightened behavioral disinhibition in pursuit of higher-intensity stimulation. Intervention strategies that upregulate everyday reward value and strengthen self-regulation may offer utility in reducing teen SU.

## Introduction

Adolescence is a developmental period characterized by a normative increase in the propensity to engage in risk-taking behaviors such as substance use (SU) (1, 2). Despite recent declines, adolescent SU remains a public health concern, with cannabis, nicotine, and alcohol being the most commonly used among youth (3, 4). Estimates from the 2025 *Monitoring the Future* study report indicate that ~16% of 10th graders used cannabis, ~15% vaped nicotine, and ~26% consumed alcohol in the year prior (5). These rates are concerning because early SU initiation confers elevated risk for later SU disorders and mental health issues (6–8). Accordingly, clarifying *neurobiological* and *behavioral* risk-resilience mechanisms is a priority to facilitate intervention refinement for preventing, delaying, or reducing teen SU.

Neurobiological models highlight the striatum as a key contributor to motivational and reinforcement processes relevant to the emergence, escalation, and maintenance of SU (9–11). The striatum encodes incentive salience and translates the value of rewarding stimuli into approach behavior (12, 13), in part via dopaminergic input from midbrain nuclei [i.e., ventral tegmental area [VTA], substantia nigra pars compacta [SNc]] (10, 14). The ventral striatum (VS; nucleus accumbens) supports incentive valuation and instrumental action-outcome learning, leveraging dopaminergic reward-prediction-error signals, predominantly from the VTA, to update expected-value representations and goal-directed motivation (15–17). The dorsal striatum (DS; caudate, putamen), which receives predominantly SNc dopaminergic input, links value to action, translating motivational information into motor preparation via cortico-striato-thalamo-cortical loops involving the premotor cortex and supplementary motor area (18–22). Both striatal subdivisions are responsive to primary and secondary rewards, to reward-predictive cues, and, among chronic users, are consistently engaged by drug-related stimuli (23–25). Individual differences in striatal activity during reward processing can be linked to real-world health-related behaviors including risk-taking, impulsive decisions, and SU outcomes (26–30). Further, chronic substance exposure is accompanied by fronto-striatal alterations contributing to blunted sensitivity to non-drug rewards and heightened responsivity to drug-related cues (11, 31, 32). Importantly, such alterations may not be solely the consequence of chronic use, as accumulating evidence suggests that reward-related brain activity among adolescents is linked with future SU, representing a plausible neurobiological risk factor for use progression (29, 33, 34).

Developmental models characterize adolescence as a period of fronto-striatal plasticity, with motivation-related limbic maturation outpacing that of control-related prefrontal systems (1, 35, 36). Within striatal circuits, synaptic pruning, dendritic spine remodeling, and changes in dopaminergic receptor expression alter how rewarding stimuli are evaluated and translated into action (37–39). These neurodevelopmental changes behaviorally coincide with heightened novelty seeking, exploration, and peer-influenced risk-taking (40–42). Against this developmental backdrop, individual differences in reward-related striatal reactivity may contribute to variability in real-world behavioral disinhibition. The Monetary Incentive Delay (MID) task is a commonly used laboratory paradigm to probe striatal and behavioral responses during different phases of reward processing (23, 43, 44). In the MID, symbolic cues are presented signaling potential gains or losses of varying magnitudes and, after an anticipation interval, a target stimulus is presented to which participants respond with a speeded button press to maximize gains and minimize losses. Behaviorally, adolescents show faster response times (RTs) and higher hit rates (HRs) to gain-trial targets (vs. loss or neutral ones), with responses scaling as incentives increase (23, 45, 46). Neurobiologically, ventral striatal activity during gain anticipation tracks cue-encoded expected value, whereas dorsal striatal (caudate) engagement relates to subsequent RT, consistent with value-to-action mapping (23, 44, 45, 47, 48). As such, the MID task may provide useful metrics to link reward-related neurobiology with individual differences in behavioral disinhibition.

Within the broader construct of disinhibition, impulsivity can be conceptualized as a proximal facet preceding externalizing and SU behaviors. Impulsivity is a relatively stable tendency toward rash, unplanned actions and difficulties delaying gratification, both reflecting reduced top-down control over reward-driven approach behaviors (1, 49). Across longitudinal cohorts, adolescents who endorse higher impulsivity exhibit greater subsequent rule-breaking, oppositionality, aggression, and SU involvement, supporting a mechanistic pathway from neurobehavioral vulnerability to later mental health and SU outcomes (50–57). These associations persist even after adjusting for demographics, familial risk, and baseline behavioral problems, suggesting that impulsivity represents a precursor to a downstream externalizing phenotype linked with SU risk. Prospectively, higher baseline impulsivity is associated with earlier SU initiation, faster escalation, and greater persistence of cannabis, nicotine, and alcohol use among adolescents (50, 52, 53, 58–60). Variation in reward-related neurobiology may shape impulsive dispositions, manifesting as blunted sensitivity to low-intensity incentives or a bias toward immediate, higher-intensity stimulation, consistent with reward-deficit and incentive-salience accounts (13, 43, 61–65). Accordingly, we conceptualized impulsivity as a broad liability serving as a proximal bridge linking reward-related striatal responsivity to future externalizing behaviors and real-world risk for cannabis, nicotine, and alcohol use.

While impulsivity may operate as an early neurobehavioral liability, externalizing symptomatology reflects a downstream mental health outcome that consolidates vulnerability and amplifies SU risk (50, 53). Externalizing symptoms (e.g., rule breaking, aggression) provide a clinically grounded index of disinhibition that prospectively predicts earlier SU onset and steeper escalation (66–68). Longitudinal work further indicates that externalizing trajectories across childhood and adolescence are among the most robust behavioral predictors of later cannabis, nicotine, and alcohol use (57, 69, 70). Neurobiologically, externalizing problems have been linked to alterations in reward- and salience-processing circuitry (e.g., striatum, insula), along with weaker recruitment of prefrontal control during inhibition and performance monitoring (71–73). Together with impulsivity, externalizing problems may capture the behavioral expression of altered reward and salience processing manifesting as heightened sensitivity to immediate incentives or reduced responsivity to low-intensity rewards that can bias some youth toward risk-taking behaviors.

An area of ambiguity in MID-based adolescent studies is whether a liability for impulsivity, externalizing problems, and SU relates to a “reward-sensitivity” (hyperresponsivity) or a “reward-deficit” (hypo-responsivity) pattern in striatal reactivity (74). Hyperresponsivity accounts suggest that stronger reward-related striatal responses heighten the motivational pull of potential rewards, fostering sensation seeking, approach behavior, and early SU involvement (29, 75–77). Hyporesponsivity accounts suggest that blunted striatal responses to low-intensity incentives predict impulsivity, externalizing symptoms, and subsequent SU by predisposing some youth to seek more potent, immediate, or higher-intensity rewards (27, 33, 46, 62, 78–81). As evidence exists for both views, the directionality of the relationship between striatal activity and measures of behavioral disinhibition may vary as a function of developmental timing, prior substance exposure, or the specific neuroimaging task implementation (74, 82). Despite divergent findings, both accounts imply that individual differences in reward-related striatal activity are behaviorally meaningful when considering adolescent mental health and SU outcomes.

Given these neurobiological and behavioral factors, we conceptualized reward-related striatal reactivity, impulsive dispositions, and externalizing tendencies as components of a mechanistic pathway to adolescent SU. We considered three primary hypotheses pertaining to task-related, brain-behavior, and SU outcomes. For task-related outcomes, we expected anticipatory, cue-related striatal activity to be greatest (and target-related RTs to be fastest) on MID gain trials relative to loss or neutral trials. We also expected behavioral responses to scale with incentive magnitude, consistent with enhanced motivation to maximize positive outcomes. For brain-behavior relationships, we expected greater cue-related striatal activity on gain trials to correlate with faster target-related RTs and self-reported impulsivity (but we did not specify the direction of this impulsivity association given mixed evidence in the literature). For SU outcomes, we expected that individual differences in baseline striatal activity and/or impulsivity would predict mental health (externalizing symptoms) and subsequent SU outcomes (cannabis, e-cigarette, alcohol) assessed at follow-up (~15 months later). To formalize this, we tested serial mediation models evaluating whether impulsive dispositions and externalizing tendencies mediated an association between striatal activity and substance-specific days of use.

## Methods

### Participants

We analyzed data from a subsample of 140 adolescents [49% female, 83% White, 88% Hispanic/Latino(a), age: 14.9 ± 0.7 years (mean ± SD), Table 1] who completed wave 1 (W1, *n* = 164) and wave 2 (W2, ~15 months later, *n* = 151) of a larger longitudinal study (*N* = 264) examining SU etiological factors. While the current analyses were limited to those participants completing MRI scans, the resulting subsample did not differ from the full cohort on any demographic or SU measures (*p’s* = 0.15–0.99). An additional 11 participants were excluded from subsequent analyses given failed MRI quality control (e.g., excessive motion, *n* = 5) or incomplete behavioral data (e.g., terminated scan earlier, *n* = 5). At enrollment, participants were high school 9th–10th graders (age range: 14–16 years) and their caregivers. W1 data collection spanned March 2018 through December 2019 and W2 from June 2019 to June 2021. Eligibility criteria for youth included English fluency and no diagnosis of a learning disorder, intellectual or physical disability, neurological condition, or severe mental illness. MRI exclusion criteria further included left-handedness, non-removable metal, claustrophobia, and pregnancy. Prior SU at enrollment was not exclusionary to increase the likelihood of the sample’s use over the study duration and to yield a more representative sample.

### Study procedures

Recruitment occurred at public schools, and caregivers of adolescents interested in participating were contacted for screening. Eligible individuals were then scheduled for an initial visit (W1a). Following consent/assent, youths and caregivers completed questionnaires in separate rooms to ensure confidentiality for ~90 min and ~45 min, respectively. For eligible participants, initial data collection also included a second visit (W1b) within one month of W1a involving additional youth questionnaires (~15 min) and a MRI scan session (~90 min). Two tasks were completed during the MRI (i.e., a working memory n-back and a MID task) with a 10-min resting-state fMRI scan between the tasks. W2 procedures mirrored W1a, however, given COVID-19 restrictions, some visits were completed remotely. Questionnaires were administered via REDCap using a tablet at in-person visits and on personal electronic devices for remote visits. Study procedures were approved by the Institutional Review Board and participants were compensated after each visit. Additional participant and procedure details are reported elsewhere (83–88).

### Self-report measures

Impulsive dispositions were characterized at W1b via the short version of the *Urgency, Premeditation, Perseverance, Sensation Seeking, Positive Urgency* (UPPS-P) behavior scale (49, 89). The UPPS-P quantifies a multidimensional propensity toward rash actions spanning emotion-driven reactivity, conscientious control capacities, and appetitive approach behaviors. Specifically, the instrument considers five facets indexing rash actions under negative affect (negative urgency), rash actions under positive affect (positive urgency), acting without forethought (lack of premeditation), difficulty sustaining effort on challenging tasks (lack of perseverance), and preference for intense/novel experiences (sensation seeking) (49, 89). Youth rated the questionnaire’s 20 items on a 4-point Likert scale (1 = strongly agree, 4 = strongly disagree). Example items include: “*I welcome new and exciting experiences and sensations, even if they are a little frightening and unconventional*” (sensation seeking), “*I tend to act without thinking when I am really excited*” (positive urgency), and “*I like to stop and think things over before I do them*” (lack of premeditation). To reduce the number of comparisons and quantify a broad multidimensional index, we summed ratings for all items (reverse-scoring where appropriate) to yield a total score with higher values indicating a more impulsive disposition (Cronbach’s *α* = 0.62). The UPPS-P exhibits a replicable five-factor structure and solid construct validity (89–91) where higher total scores have been linked with externalizing-related phenotypes in large youth cohorts (90, 92) and with risky behaviors, including SU, among young adults (89). Accordingly, we conceptualized total scores as a parsimonious index relevant to mental health and SU outcomes.

Mental health and SU outcomes were characterized at W2 which occurred ~15 months after W1b. Mental health characteristics were quantified with the *Achenbach System of Empirically Based Assessment (ASEBA) Youth Self-Report* (YSR) (93). Specifically, we focused on the externalizing composite consisting of the rule-breaking and aggressive behavior subscales (Cronbach’s *α* = 0.83). To minimize overlapping information (i.e., multicollinearity) with SU measures, we removed three SU items from the rule-breaking subscale (66, 94). SU was assessed at W1a (baseline) and W2 (follow-up) using items adapted from the *Population Assessment of Tobacco and Health* (PATH) Survey (95). SU characteristics included use endorsement, days of use, and age of first use (Supplementary Table S1). We focused on cannabis, e-cigarette, and alcohol, utilizing days of use as our primary variable of interest, given these are the most commonly used substances among teens (5). Participants were asked “[*In the past year (baseline)/Since your last visit (follow-up)], on how many days did you [use cannabis/use an Electronic Nicotine Delivery System product/have one or more alcoholic drinks]?”*

### Monetary incentive delay (MID) task

To assess brain and behavioral responsivity to varying incentives, participants completed a MID task (23) version involving separate valence (i.e., gain, loss, neutral) and magnitude cues (i.e., small, medium, large) (96, 97). Participants’ overall goal was to maximize gains and minimize losses by responding as quickly as possible when a visual target (white cross) appeared (Supplementary Figure S1). Each MID trial consisted of four stimuli: a valence cue (350 ms; blue circle = gain, red square = loss, yellow triangle = neutral), a magnitude cue (400 ms; small, medium, large), a speeded target (variable duration), and performance feedback (1,500 ms). Two variable interstimulus intervals (ISI, 800–3,200 ms of fixation) separated the valence and magnitude cues, and the magnitude cues and target stimuli such that their durations summed to 4,000 ms. A variable intertrial interval (ITI, 1,600–4,800 ms of fixation) separated the current trial’s feedback display from the next trial’s initial valence cue. To introduce additional temporal jitter, null trials (3,200–4,800 ms of fixation, *n* = 64) were interleaved throughout the task. Targets were presented with a variable duration initialized at 350 ms and adjusted dynamically in 25 ms steps. The target response window was narrowed after hits and widened after misses to maintain ~66% accuracy. Feedback displayed the single-trial outcome and a running total of money accumulated over the task. To balance affective responding across valence, monetary magnitudes were asymmetric where gain trials offered +$2.50 (small), +$10 (medium), and +$15 (large) incentives and loss-trial incentives were −$1.50, −$6, and −$9. On gain trials, a target hit (i.e., a response during the target’s display window) yielded the gain magnitude amount (e.g., small: +$2.50) and misses yielded a base increase of +$1. On loss trials, hits incurred a base decrease of −$0.75 and misses incurred the loss magnitude amount (e.g., large: −$9). No incentives were involved on neutral trials corresponding to +$0 feedback for both target hits and misses. Participants were instructed and trained to respond to all targets with a right index finger button press and practiced in a mock scanner.

The task included 168 trials across four, 8-min runs composed of 72 gain (42.9%), 72 loss (42.9%), and 24 neutral trials (14.3%). Gain and loss trials were each subdivided into 24 small, 24 medium, and 24 large magnitude trials. E-Prime (Psychology Software Tools) controlled stimulus presentation and recorded responses. MID incentives were fictitious as compensation was fixed and not contingent on overall performance. Behavioral measures were mean target-related response times (RTs) and hit rates (HRs) as a function of valence and magnitude cues. Mean RTs were computed for correct trials only, including late responses and excluding early responses. HRs were the percentage of on-time responses during the target display window within each cue condition.

### MRI data

Data were acquired with a 3 T Siemens Prisma scanner. During the MID task, sixty 2.4-mm thick slices were collected with a multiband gradient-echo, echo-planar imaging sequence sensitive to blood oxygenation level-dependent (BOLD) effects [repetition time (TR) = 800 ms; echo time (TE) = 30 ms; flip angle (FA) = 52 °; field of view (FOV) = 216 mm]. High-resolution T1-weighted structural images were obtained using a magnetization-prepared rapid gradient-echo sequence (TR = 2,500 ms; TE = 2.9 ms; FA = 8 °; voxel size = 1 mm^3^). MRI data were preprocessed with *fMRIPrep* 20.2.1 (98, 99) and analyzed in AFNI (100) following recommended best practices (101, 102). Preprocessing steps included skull-stripping, distortion correction, co-registration, motion correction, slice-time correction, tissue segmentation, and spatial normalization (Supplementary Text).

After preprocessing, MID runs were de-meaned and submitted to subject-level, voxel-wise multiple regression with AFNI’s *3dDeconvolve* and *3dREMLfit* (v20.2.10). Task regressors modeled anticipatory *valence cues* (gain, loss, neutral), *magnitude cues* (small, medium, large, zero), and feedback (hit, miss) (96, 97). Regressors were delta functions convolved with a canonical hemodynamic response and its temporal derivatives. Nuisance regressors included six motion parameters, censored time points, and scanner drift estimates. For each task regressor, voxel-wise amplitudes (*β*’s) were expressed as percent signal change from baseline. To increase sensitivity while controlling for family-wise error, we conducted small-volume corrected (SVC) analyses as opposed to whole-brain analyses. Specifically, we considered only those voxels within a meta-analytically defined mask generated via Neurosynth (103) for the term “reward processing” (Supplementary Figure S2). Second-level analyses using AFNI’s 3dMVM (104) focused on anticipatory valence contrasts (i.e., gain vs. neutral and loss vs. neutral) and included age, biological sex, and mean framewise displacement as covariates. Group maps for gain and loss contrasts were thresholded at *p*_*corrected*_ < 0.01 (*p*_*voxel*−*wise*_ < 0.001, cluster extent: 11 voxels, *3dClustSim* with spatial autocorrelation correction). Mean *β*-values were extracted from significant clusters within the SVC search mask for visualization and follow-up assessments in R (v4.2.1) (105).

### Statistical analyses

For task behavioral measures, repeated-measures ANOVA examined cue-related effects on target-related RTs and HRs. Separate ANOVAs considered valence (gain vs. loss vs. neutral) and magnitude cue effects (small vs. medium vs. large within gains and losses) including age, sex, race, and ethnicity as covariates and Bonferroni-corrected follow-up comparisons (*n* = 3). For brain-behavior associations, bivariate Pearson correlations were used to characterize interrelations between anticipatory cue-related brain activity and objective task performance, as well as subjective impulsivity. For substance use outcomes, we first summarized descriptives and bivariate correlations between study variables to facilitate covariate selection (Supplementary Tables S1–S2) which included age, sex, race, ethnicity, and substance-specific days of use at W1. Three separate models assessed whether impulsivity (M_1_: UPPS-P total scores) and/or externalizing (M_2_: YSR) mediated the impact of anticipatory striatal activity (X: gain-cue *β*) on cannabis, e-cigarette, or alcohol use (Y: days of use). We considered each SU outcome separately (as opposed to modeling SU more broadly) to empirically determine whether risk pathways are shared or substance specific. Parameter estimates and 95% confidence intervals (95%CIs) were derived via 5,000 bootstrap samples using PROCESS v4.2 (106) for IBM SPSS Statistics (v30).

## Results

### MID behavioral outcomes

**FIGURE 1:**
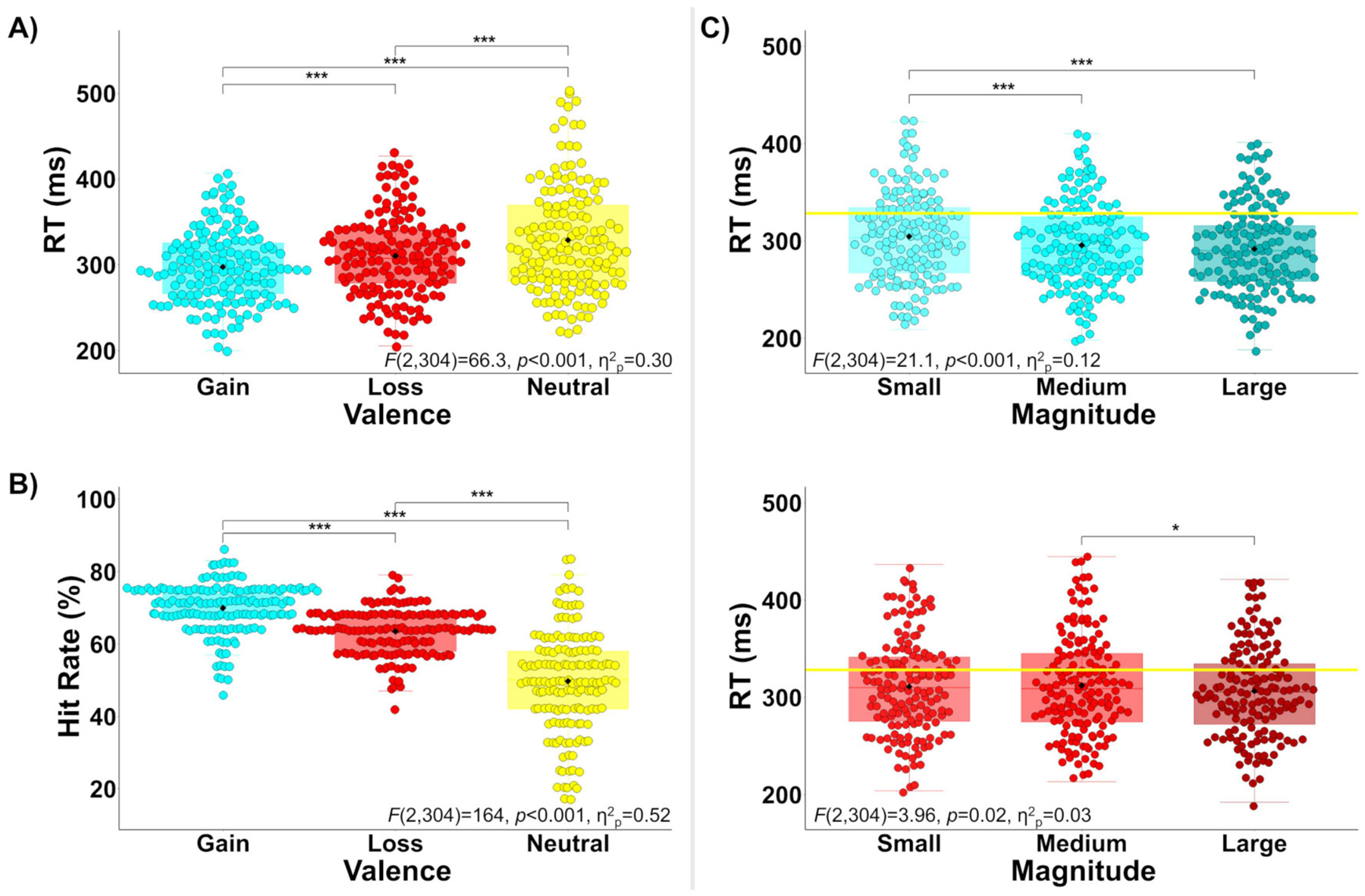
MID behavioral performance. **(A)** Target-related response times (RTs) were fastest for gain trials [vs. loss: *t*[152]=−9.04, *p* < 0.001; vs. neutral: *t*[152]=−9.62, *p* < 0.001] and slowest for neutral trials [vs. loss: t(152)=−5.88, *p* < 0.001]. **(B)** Similarly, target-related hit rates (HRs) were highest for gain trials [vs. loss: *t*[152] = 9.01, *p* < 0.001; vs. neutral: *t*[152] = 15.4, *p* < 0.001] and lowest for neutral trials [vs. loss: *t*(152 = 10.5, *p* < 0.001). **(C)** When considering gain trials (top, cyan), RTs were fastest for large gains [vs. small: *t*(152) = 6.26, *p* < 0.001] and slowest for small gains [vs. medium: *t*(152) = 4.52, *p* < 0.001]. When considering loss trials (bottom, red), RTs were fastest for large losses [vs. medium: t(152) = 2.47, *p* = 0.04] with no difference between medium and small losses. For reference, the horizontal yellow line indicates average target-related RT on neutral trials. *p < 0.05, ***p < 0.001.

Repeated-measures ANOVA examined anticipatory cue-related valence effects on target-related performance (Figure 1). As hypothesized, RTs were fastest on gain trials, intermediate on loss, and slowest on neutral trials [Figure 1A, *F*(2,304) = 66.3, *p* < 0.001]. HRs followed a similar pattern [Figure 1B, *F*(2,304) = 164, *p* > 0.001], indicating enhanced behavioral motivation following gain and loss cues relative to neutral ones. Notably, neutral trial HRs showed high variability, suggesting that some participants selectively withheld responses, despite instructions to respond to all targets. While trial outcomes were contingent on target-related performance for gains and losses (together ~86% of trials), no money was at stake on neutral trials (~14%). Given this lack of incentivization, “oddball” neutral cues likely served as a salient signal for some participants to modify behavioral output (i.e., withhold a response).

Regarding cue-related magnitude effects for gains, target RTs decreased with increasing reward value, such that RTs were fastest on large, intermediate on medium, and slowest on small incentive trials [Figure 1C, top; *F*(2,304) = 21.1, *p* < 0.001]. For losses, RTs were fastest following large magnitude cues with no difference between small and medium cues [Figure 1C, bottom; *F* (2,304) = 3.96, *p* = 0.02]. These outcomes were consistent with the expected enhancement of behavioral motivation with larger incentives.

### MID brain outcomes

**FIGURE 2:**
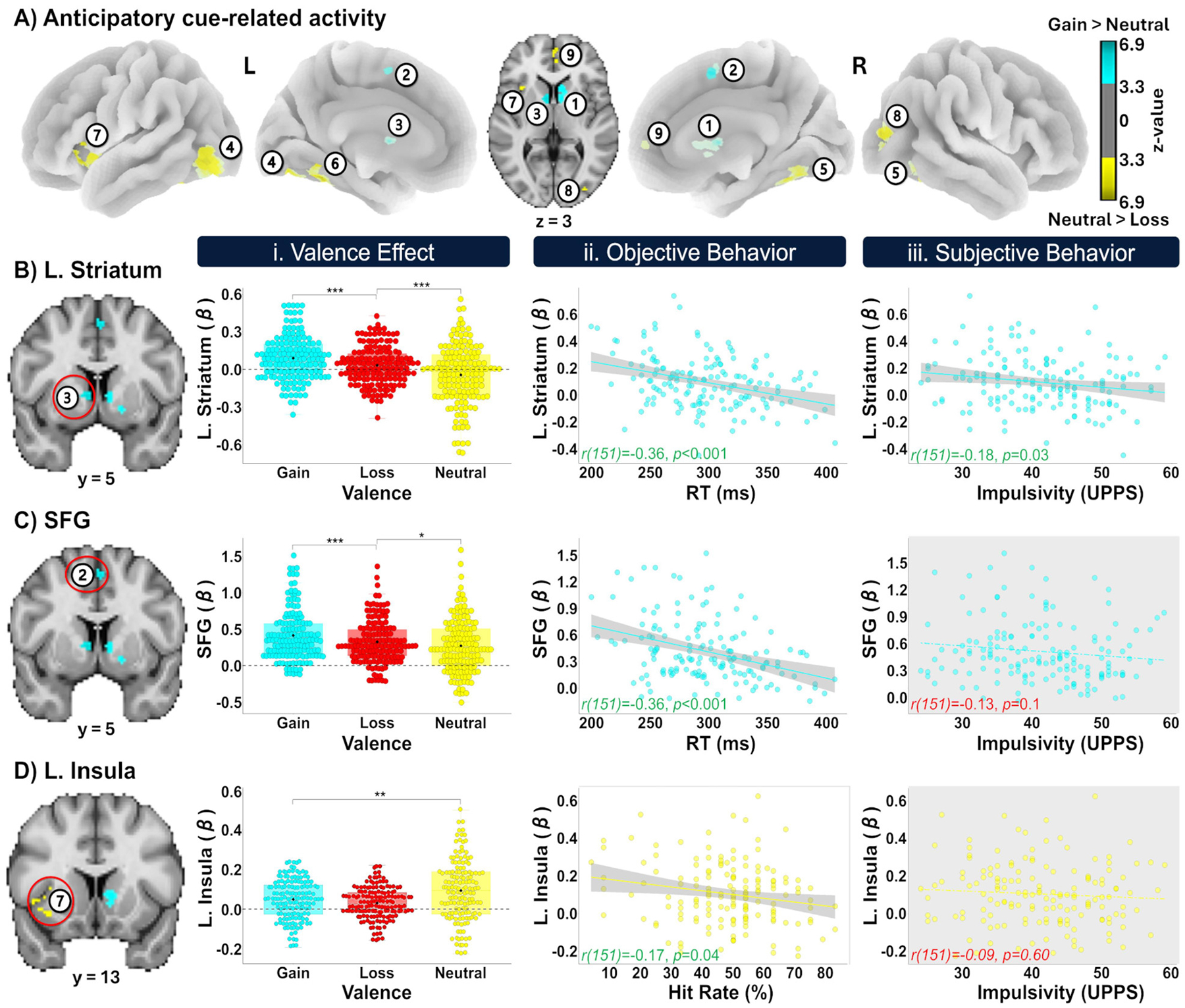
MID anticipatory cue-related brain activity and brain-behavior relations. **(A)** Clusters showing greater activation following gain cues (vs. neutral, cyan) and neutral cues (vs. loss, yellow, *p_corrected_* < 0.01, see Table 1 for cluster coordinates). **(B)** Left striatal activity showed hypothesized valence effects and correlations with task performance and self-reported impulsivity. Regarding valence effects **(i)**, striatal activity was greater following gain versus loss cues [*t*(152) = 3.34, *p* = 0.003, Bonferroni-corrected], and greater following loss versus neutral cues [*t*(152) = 3.27, *p* = 0.002, Bonferroni-corrected]. We note that these two selective *post-hoc* comparisons were independent of the voxel selection criteria. Regarding performance (**ii**), left striatal activity negatively correlated with target-related RT, as did right striatal activity [*r*(151)=−0.29, *p* < 0.001, data not shown]. Regarding impulsivity (**iii**), left striatal activity negatively correlated with UPPS total scores whereas right striatal activity did not [*r*(151)=−0.10, *p* = 0.4, data not shown]. Our MID task implementation required a right-handed button press suggesting that the more robust left striatal correlation with impulsivity may relate to action execution lateralization. **(C)** Speaking to regional specificity, superior frontal gyrus (SFG) activity also showed similar valence effects **(i)** and relations with task performance (**ii**), but not impulsivity (**iii**). **(D)** In contrast, left insula activity displayed a different pattern for valence effects and brain-behavior relations. Insula activity was greater following neutral (vs. gain) cues [**i**, *t*(152)=−3.62, *p* = 0.001, Bonferroni-corrected] in the absence of a difference between gain and loss cues [*t*(152) = 1.92, *p* = 0.17]. Insula activity did not correlate with RT [*r*(151) = 0.12, *p* = 0.3, data not shown] nor impulsivity (**iii**). Rather, insula activity negatively correlated with neutral-trial target HR (**ii**). ** *p* < 0.01, *** *p* < 0.001.

SVC analyses identified distinct patterns of anticipatory cue-related brain activity (Figure 2; Table 2). Gain (vs. neutral) cues elicited greater activation in the bilateral striatum (caudate) and superior frontal gyrus (SFG, supplementary motor area) (Figure 2A), consistent with reward anticipation and behavioral motivation. Neutral (vs. loss) cues elicited greater activation in the left insula, dorsomedial prefrontal cortex (PFC), and occipital regions, a pattern consistent with the detection of infrequent, behaviorally salient oddball stimuli rather than valence processing. Follow-up analyses interrogating significant clusters confirmed that left striatal (Figures 2Bi) and SFG activations (Figures 2Ci) were highest following gain, intermediate after loss, and lowest following neutral cues, mirroring behavioral patterns. In contrast, left insula activation was highest following infrequent neutral cues, with no difference between gain and loss trials (Figures 2Di), consistent with a role in the detection of salient oddball events.

### Brain-behavior relations

Correlation analyses considered associations between cue-related brain activity, task performance, and self-report measures. Greater left striatal activation to gain cues correlated with faster RTs to subsequent targets [Figures 2Bii, *r*(107)=−0.36, *p* < 0.001] and lower self-reported impulsivity [Figures 2Biii, r(107)=−0.18, *p* = 0.03] but was unrelated to target HR [*r*(107) = 0.03, *p* = 0.7]. SFG activation to gain cues showed a similar association with RT (Figures 2Cii), but not with impulsivity (Figures 2Ciii). In contrast, greater insula activation following neutral cues correlated with lower HRs to subsequent targets [*r*(107)=−0.17, *p* = 0.04], but not with RT [*r*(107) = 0.12, *p* = 0.3] or impulsivity [*r*(107)=−0.09, *p* = 0.6]. This HR association aligns with the insula’s role in salience detection and modulating response strategies.

### Substance use outcomes

**FIGURE 3:**
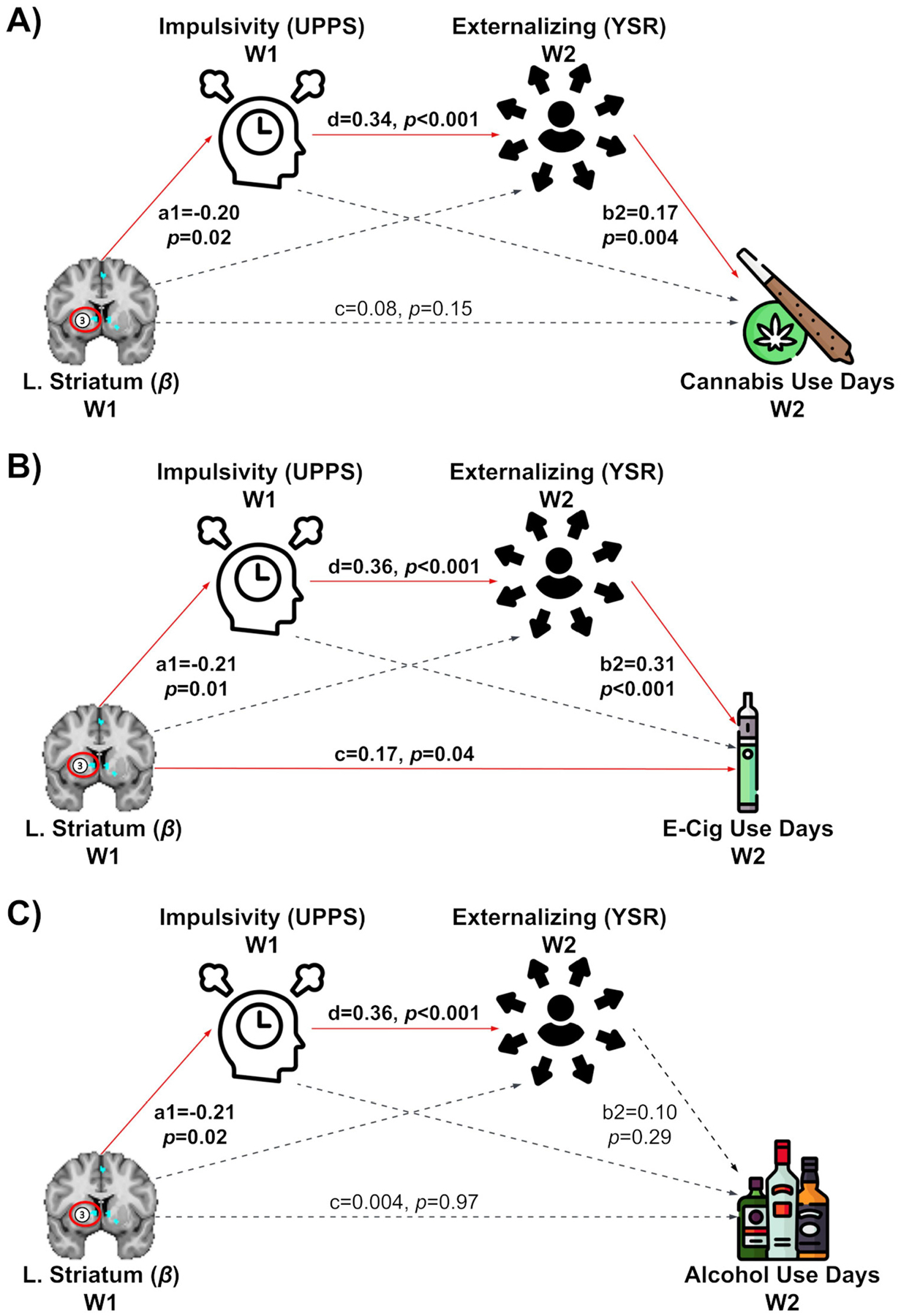
Serial mediation models linking left striatal (caudate) activity, impulsivity, externalizing, and future substance use. **(A)** Less striatal activity during gain anticipation at Wave 1 (W1) was indirectly associated with more cannabis use days at W2 via elevated impulsivity and externalizing symptomatology [indirect effect = −0.01, *95%CI* (–0.03, −0.001), Supplementary Table S3]. **(B)** A similar indirect path was observed for e-cigarette use [indirect effect = −0.02, *95%CI*(–0.06, −0.003), Supplementary Table S4], with an additional direct effect of striatal activity on use (*c* = 0.17, *p* = 0.04) indicative of partial mediation (see: Supplementary Table S4 for further discussion). **(C)** In contrast and suggesting substance specificity, no indirect or direct effects linked striatal activity with alcohol use (Supplementary Table S5). Standardized path coefficients are shown.

Separate serial mediation models examined the degree to which left striatal activity during gain anticipation predicted future cannabis, e-cigarette, and alcohol use via impulsivity and externalizing problems while controlling for age, biological sex, race, ethnicity, and substance-specific baseline use (Figure 3). For cannabis use (Figure 3A), a significant serial indirect effect was observed (Supplementary Table S2) such that less striatal activity predicted more use days at follow-up through impulsivity and externalizing [indirect effect: −0.01, *95%CI* (−0.03, −0.001), *R*^*2*^ = 0.62, Supplementary Table S3]. Specifically, less striatal activity was linked with more impulsivity, which in turn predicted higher externalizing scores, which then linked with more cannabis use. No effects were detected linking right striatal activity and cannabis use (Supplementary Table S3; Supplementary Figure S3). For e-cigarette use (Figure 3B), a similar pathway was observed for the left striatum [indirect effect = −0.02, *95%CI* (–0.06, −0.003), *R*^*2*^ = 0.18], with an additional direct effect [direct effect = 0.17, *95%CI* (0.005, 0.33), *p* = 0.04, Supplementary Table S4] linking more striatal activity to more e-cigarette use, suggesting partial mediation. Again, no effects emerged for the right striatum (Supplementary Table S4; Supplementary Figure S3). For alcohol use (Figure 3C), neither indirect nor direct effects were significant for either the left or right striatum (Supplementary Table S5; Supplementary Figure S3). These outcomes remained unchanged when also utilizing W1 days of use for all three substances as covariates (Supplementary Tables S6–S8).

## Discussion

We considered whether individual differences in reward-related striatal activity and impulsivity among adolescents predicted mental health (externalizing symptoms) and SU outcomes (cannabis, nicotine, alcohol) over a year later. Using a MID task, we observed expected activation of the bilateral striatum and SFG following anticipatory gain cues, reflecting recruitment of dopaminergic circuitry supporting reward-driven motivation and goal-directed behaviors (96, 97, 108). In contrast, insula activation following neutral cues appeared to reflect detection of infrequent, yet behaviorally salient, oddball events rather than valence processing (109–111). Across participants, *more* striatal activation to gain cues was associated with enhanced target-related performance (i.e., faster RTs) and *lower* self-reported impulsivity. Importantly, serial mediation models further indicated that *less* striatal activity during reward anticipation indirectly predicted more cannabis and e-cigarette use (but not alcohol) at follow-up via *higher* impulsivity and externalizing symptoms. Taken together, these findings suggest that blunted striatal activity reflects reduced motivational drive for normative or lower-intensity rewards (e.g., fictitious MID monetary rewards), which may contribute to SU vulnerability via heightened behavioral disinhibition in pursuit of higher-intensity stimulation.

Our MID task implementation yielded expected patterns of behavioral and brain outcomes across cue conditions. Behaviorally, target RTs were fastest and HRs highest on gain trials, intermediate for losses, and slowest/lowest for neutral trials, indicative of enhanced behavioral motivation in the presence of performance-contingent incentives. Performance also scaled with cue magnitude (i.e., faster RTs for larger vs. smaller gains/losses), indicating that increased incentive value further enhanced motivated responding. These outcomes confirmed that participants engaged with the task as intended, and that the task’s incentive structure yielded graded behavioral responsivity to cue manipulations. Neurobiologically, gain cues elicited striatal and SFG (supplemental motor area) activation paralleling behavioral outcomes. Indeed, greater dorsal striatal activation during gain anticipation correlated with faster target RTs, suggesting that heightened reward-related signaling facilitated motor readiness and goal-directed performance. Although the ventral striatum is typically linked with reward valuation and motivation, engagement of the caudate aligns with its role translating incentive cues into actions (47). The caudate receives convergent dopaminergic input from both the VTA and SNc, thereby serving as an integrative hub where motivational and motor signals intersect to guide actions toward positive outcomes (18, 20, 22, 112). Therefore, we suggest that adolescents showing *stronger* dorsal striatal engagement more efficiently mobilized motor responses in the service of MID reward acquisition, reflecting effective integration of motivational signaling and action execution (113, 114).

On the other hand, *weaker* engagement of striatal circuitry has been linked with developmental liability to disinhibitory behaviors and SU. For example, reduced striatal activity among adolescents has been associated with elevated impulsivity and externalizing symptoms (27, 46, 62, 78–81). Prospective studies likewise indicate that less striatal responsivity during reward anticipation can predict subsequent SU and related problem behaviors (27, 33, 34). We speculate that adolescents showing blunted striatal responsivity to MID gain cues may derive less motivational value from low-intensity rewards (e.g., fictitious monetary incentives, academic achievement, social approval), thereby heightening the drive for higher-intensity or pharmacological stimulation. This account parallels neurobiological models in which chronic drug exposure further dysregulates reward circuitry, promoting compulsive drug-seeking and -taking (115, 116) and the incentive-sensitization theory, positing that repeated use progressively hijacks motivational systems (13). Although these frameworks describe neuroadaptations among established users, analogous inefficiencies in reward signaling during early stages of adolescence SU may confer risk by enhancing motivation for stronger stimulation, thereby setting the stage for future externalizing behaviors and SU escalation (50).

While our outcomes align with a hypo-responsivity account, the adolescent reward processing literature is heterogenous, with reports of both blunted and heightened striatal responses being tied to impulsivity, externalizing symptoms, or SU (74). Multiple longitudinal studies suggest that more striatal activity during reward anticipation predicts subsequent SU initiation among substance-naïve adolescents, and that greater responsivity can be seen among youth high (vs. low) in impulsivity/novelty seeking (29, 75–77). Several non-mutually exclusive factors may account for why disinhibitory behaviors have been linked with both striatal hypo- and hyperreactivity. These include the reward-processing phase examined (anticipation vs. outcome), the anatomical loci under consideration (dorsal vs. ventral striatum), prior substance exposure (naïve vs. experienced), and specific task implementations and demands (e.g., speeded responding, feedback timing) (74, 82). Developmental timing may also contribute to this heterogeneity, as motivation-related dopamine systems mature earlier than prefrontal control regions, creating an imbalance that can transiently amplify striatal reactivity during adolescence (1, 117).

From a hypo-reactivity perspective, our serial mediation analyses suggest one possible mechanistic pathway linking lower striatal responsivity during reward anticipation with future cannabis and e-cigarette use indirectly via impulsivity and externalizing symptoms. Neurobiologically, these associations align with evidence implicating dorsal striatal function as a neural correlate of impulsivity (118) and with pharmacological PET findings linking reduced dopaminergic signaling with greater disinhibition (64, 119–121). To the extent that striatal BOLD activity mirrors phasic dopamine release (122, 123), the blunted activation observed here may reflect attenuated dopaminergic signaling contributing to less motivational drive for lower-intensity incentives. Within a canonical inverted-U framework linking individual differences in dopamine signaling and behavior, lower dopamine levels are associated with slower value-to-action mapping, reduced responsiveness to low-intensity rewards, and poorer inhibitory control (21, 124–126). Consistent with this framework, psychostimulants (e.g., methylphenidate) enhance fronto-striatal catecholamine transmission and often reduce externalizing symptoms in people diagnosed with ADHD, likely by shifting low-dopamine states toward a more optimal range on an inverted-U (127–129). Taken together, striatal hypoactivation may reflect a dopamine-related motivational inefficiency that, for some adolescents, elevates SU risk indirectly via disinhibitory tendencies and a bias toward higher-intensity stimulation.

Substance-specific patterns in our mediation models suggested that this indirect pathway preferentially predicted cannabis and e-cigarette outcomes, but not alcohol. Although all substances of abuse can increase mesocorticolimbic extracellular dopamine (116, 130), the route and speed of drug delivery, receptor targets, and net dopaminergic impact can differ. Pharmacodynamically, cannabis and nicotine engage mesocorticolimbic circuitry in ways that more closely map onto the anticipatory striatal signaling implicated here. That is, nicotine directly excites VTA dopamine neurons via nicotinic acetylcholine receptors, producing reliable phasic dopamine release in the striatum, whereas THC disinhibits midbrain dopamine neurons via cannabinoid (CB1) receptors on GABAergic terminals, yielding modest but consistent dopamine elevations (6, 131–135). By contrast, alcohol engages GABAergic, glutamatergic, and opioidergic mechanisms with slower, more variable dopaminergic consequences (116, 130, 136). Evidence from lesion and pharmacological models also indicates that alcohol reinforcement can be sustained through non-dopaminergic mechanisms to a greater degree compared to cannabis or nicotine (137–140). Also, inhalation (smoking/vaping) provides rapid CNS drug delivery and associated phasic reinforcement, features that may be particularly consequential for adolescents with blunted anticipatory striatal responses. Psychologically, the absence of an externalizing-mediated pathway suggests an alternative route to alcohol use. One less common but well-supported pathway to adolescent SU, particularly alcohol, involves internalizing processes (e.g., negative affect, anxiety, depressive symptoms) operating via negative reinforcement mechanisms, rather than externalizing processes via positive reinforcement (141–143). Socially, cannabis, nicotine, and alcohol use are also strongly shaped by peers, substance availability, and social norms, with alcohol use often perceived as more normative (66, 144, 145). In a broader biopsychosocial context, we suggest that reduced striatal responsivity may reflect a neurobiological vulnerability that amplifies risk when use opportunities arise, setting the stage for SU trajectories that are further shaped by other psychological and social factors.

Our findings are timely given technological and societal shifts that are rapidly transforming the adolescent SU landscape. For example, legalization, commercialization, and the proliferation of high-potency products (e.g., concentrates) and delivery systems (vaping) have increased cannabis availability while lowering perceived risk among youth (146). Similarly, sleek device designs, appealing flavors, and targeted marketing have fueled e-cigarette experimentation among teens, while evolving technology has increased nicotine yields and addiction liability (147). For today’s youth these shifts coincide with a developmental period characterized by heightened neuroplasticity, social influence, and reward sensitivity amplifying vulnerability. As such, delineating brain and behavioral markers linked with cannabis and e-cigarette use, such as blunted striatal activity and disinhibitory tendencies, remains a public health priority to inform selective prevention and early intervention. Such markers may help identify youth most susceptible to the reinforcing, addiction-forming effects of early cannabis and nicotine exposure and guide interventions that strengthen reward motivation and self-regulation.

Adolescence is a critical period for the maturation and calibration of reward and control systems, making it both a window of elevated SU risk and an opportunity to shape long-term motivation and self-regulation. During this period, dopaminergic signaling and fronto-striatal connectivity are highly plastic and sensitive to environmental influences, such that experiences amplifying or reducing reward responsivity can have enduring behavioral consequences (27, 35). In our data, blunted anticipatory striatal activity indirectly predicted subsequent cannabis and e-cigarette use via higher impulsivity and externalizing symptoms, pointing to at least two intervention targets. First, strategies augmenting striatal activity to everyday positive experiences may promote SU resilience. Reward savoring refers to a set of skills for noticing, prolonging, and mentally enriching positive experiences so they become more reinforcing over time (148–150). Such skills are thought to increase the subjective value of low-intensity, normative rewards, helping to counter a bias toward high-stimulation options (107, 148, 150). Savoring and related mindfulness-based practices have been linked to changes in striatal and prefrontal function and associated behavioral gains, such as more positive affect, reduced drug cravings among users, and increased engagement in healthy activities (151–155). Second, strategies to strengthen intentional and controlled decision-making may also promote SU resilience. Cognitive Behavioral Therapy approaches can enhance skills to modify automatic response patterns, increase awareness of high-risk situations, and deploy delay/avoid/reframe strategies (156, 157). Such skills can provide youth with tools that facilitate more optimal health-related decision-making possibly by strengthening prefrontal control systems (32, 140, 158). Together, savoring (to upregulate adaptive reward processing) and CBT strategies (to downshift impulsive decision-making) may target mechanisms highlighted by our externalizing pathway in the service of reducing future cannabis and e-cigarette use.

Beyond gain-cue striatal responsivity, we also considered brain-behavior relations between insula activity to neutral cues and subsequent target-related responding. Specifically, more insula activity correlated with lower target HRs, which we interpreted as the selective withholding of responses on neutral trials by some participants. This brain-behavior association is consistent with the anterior insula’s role, along with the dorsomedial PFC, in the detection of goal-relevant, salient events and deployment of control-related process to modify action execution (111, 159, 160). More broadly, neutral cues elicited greater activation in the insula, dorsomedial PFC, and occipital regions, a pattern of brain activity consistent with the detection of infrequent, oddball stimuli (109–111). Importantly, neutral-cue insula responsivity did not correlate with impulsivity, suggesting regional specificity when juxtaposed with the indirect pathway linking striatal activity and SU outcomes through impulsivity and externalizing symptoms.

Our study should be contextualized by its limitations. First, our serial mediation models specified a directional chain with brain activity preceding impulsivity, bidirectional or alternative orderings remain plausible (see: Supplementary Text for additional *post-hoc* sensitivity analyses). Future work including an additional timepoint to account for temporal precedence is important. Related, our results suggest only one possible pathway to SU and we acknowledge that other unmodeled factors (e.g., internalizing, addiction severity, social influences) may represent other plausible mechanistic paths. Second, our findings may not generalize to all adolescents as our sample was predominantly Hispanic/Latina(o) teens who may differ from other ethnicities in regards to impulsivity characteristics (161), externalizing symptoms (162), and cannabis (163, 164) or e-cigarette use patterns (165), which may further be shaped by other contextual factors (166). Third, follow-up (W2) data collection took place during implementation of COVID-19 social distancing and remote learning practices, a period also involving shifting regulatory policies (e.g., Tobacco21 in December 2019). Such practices and policies have been linked with reduced adolescent SU rates (ref) (167, 168)which have persisted through 2024 (5). Fourth, reliance on self-report measures of impulsivity, externalizing, and SU behaviors introduces potential for reporting inaccuracies resulting from recall and social-desirability biases or question misunderstanding (169, 170). Lastly, although we covaried for baseline use, assessing youth prior to any substance initiation remains important for large, multisite studies (171).

This study identified one possible mechanistic pathway linking blunted reward-related striatal activity with greater cannabis and e-cigarette use over a year later via higher impulsivity and externalizing symptoms. We suggest that striatal hypoactivity reflects a dopamine-related motivational inefficiency that biases some youth toward higher-intensity stimulation, thereby increasing SU risk. By clarifying the links between brain-based reward processing and disinhibited behavioral tendencies, these findings highlight the potential utility of interventions to increase the subjective value of everyday positive experiences and strengthen intentional decision-making for reducing teen cannabis and e-cigarette use.

## Supplementary Material

Supplemental Material

The Supplementary Material for this article can be found online at: https://www.frontiersin.org/articles/10.3389/fradm.2026.1741884/full#supplementary-material

## Figures and Tables

**TABLE 1:** Participant demographic characteristics.

Characteristic	Wave 1 (*n* = 153)	Wave 2 (*n* = 140)
Age *M* ± *SD* (range)	14.9 ± 0.7 (14–16)	16.2 ± 0.7 (15–17)
**Grade level (*n*)**		
Freshmen	95	0
Sophomore	58	62
Junior	–	71
Senior	–	7
Biological Sex (Female/Male)	71/82 (46% Female)	68/72 (49% Female)
Hispanic/Latina(o) (no/yes)	25/128 (84% Hispanic)	17/123 (88% Hispanic)
**Race (*n*)**		
Asian	3 (2.0% Asian)	3 (2.1% Asian)
Black or African American	13 (8.5% Black)	11 (7.9% Black)
Multiracial*	10 (6.5% Multiracial)	10 (7.1% Multiracial)
White	127 (83% White)	116 (82.9% White)
**Caregiver SES**		
Total Household Income^a^	$50,000—$749,999	$50,000—$749,999
Highest Level of Education^b^	Bachelor’s Degree	Bachelor’s Degree

* Multiracial identity included *n* = 1 identifying as Asian and Black (0.7%), *n* = 2 identifying as Asian and White (1.4%), *n* = 4 identifying as Black and White (2.9%), and *n* = 3 identifying as Multiracial without specifying (2.1%).

a Median total household income in the past 12 months before assessment.

b Median highest level of education reported by caregivers. Study retention rate from Wave 1 (March 2018 to December 2019) to Wave 2 (June 2019 to June 2021) was 91.5%.

**TABLE 2:** MID anticipatory cue-related brain activity: cluster coordinates.

Contrast	Cluster	Region	Voxels	X	Y	Z
Gain >Neutral	1	R. Caudate, Putamen	158	9.4	10.2	1.2
	2	B. Superior Frontal Gyrus	28	1.0	4.1	54.7
	3	L. Caudate	27	−8.0	5.4	3.4
Neutral >Loss	4	L. Inf. Occipital Gyrus (BA 19)	240	−41.6	−75.9	−10.6
	5	R. Fusiform Gyrus (BA 37)	201	37.7	−57.1	−15.8
	6	L. Fusiform Gyrus (BA 37)	123	−37.3	−53.0	−18.9
	7	L. Insula (BA 13)	74	−35.2	16.3	−2.4
	8	R. Middle Occipital Gyrus	60	34.0	−84.5	12.2
	9	R. Middle Frontal Gyrus, ACC	53	0.5	52.9	2.3

Clusters are visualized in Figure 2 and coordinates (X, Y, Z) are center of mass in MNI space. B, bilateral; BA, Brodmann area; ACC, anterior cingulate cortex.

## Data Availability

The raw data supporting the conclusions of this article will be made available by the authors, without undue reservation.

## References

[1] Casey BJ, Getz S, Galvan A. The adolescent brain. Dev Rev. (2008) 28(1):62–77. doi: 10.1016/j.dr.2007.08.003 18688292 PMC2500212

[2] Miech RA, Johnston LD, Patrick ME, O’Malley PM, Bachman JG. Monitoring the future national survey results on drug use, 1975–2023: overview and detailed results for secondary school students. Ann Arbor, MI: Institute for Social Research, University of Michigan (2024). p. 75. Available online at: https://monitoringthefuture.org/results/annual-reports/

[3] Substance Abuse and Mental Health Services Administration. Key substance use and mental health indicators in the United States: Results from the 2022 National Survey on Drug Use and Health Center for Behavioral Health Statistics and Quality, Substance Abuse and Mental Health Services Administration; (2023). Contract No.: HHS Publication No. PEP23–07–01–006. Available online at: https://www.samhsa.gov/data/report/2022-nsduh-annual-national-report

[4] World Health Organization. Comprehensive Mental Health Action Plan 2013–2030. Geneva: World Health Organization (2021).

[5] Miech RA, Johnston LD, Patrick ME, O’Malley PM. Monitoring the Future Study Annual Report. National Survey Results on Drug Use, 1975–2024: Overview and Detailed Results for Secondary School Students. Ann Arbor, MI: Institute for Social Research, University of Michigan (2025).

[6] Connor JP, Stjepanovi D, Foll L, Hoch B, Budney E, Hall AJ, Cannabis use and cannabis use disorder. Nat Rev Dis Primers. (2021) 7(1):16. doi: 10.1038/s41572-021-00247-4 33627670 PMC8655458

[7] Behrendt S, Wittchen H-U, Höfler M, Lieb R, Beesdo K. Transitions from first substance use to substance use disorders in adolescence: is early onset associated with a rapid escalation? Drug Alcohol Depend. (2009) 99(1–3):68–78. doi: 10.1016/j.drugalcdep.2008.06.014 18768267

[8] Stone AL, Vander Stoep A, McCauley E. Early onset substance use in adolescents with depressive, conduct, and comorbid symptoms. J Early Adolesc. (2016) 36(6):729–53. doi: 10.1177/0272431615586463

[9] Yager LM, Garcia AF, Wunsch AM, Ferguson SM. The ins and outs of the striatum: role in drug addiction. Neuroscience. (2015) 301:529–41. doi: 10.1016/j.neuroscience.2015.06.033 26116518 PMC4523218

[10] Koob GF, Kandel DB, Baler RD, Volkow ND. Neurobiology of addiction. In: Tasman A, Riba MB, Alarcón RD, Alfonso CA, Kanba S, Lecic-Tosevski D, , editors. Tasman’s Psychiatry. Cham: Springer; (2023). p. 1–51.

[11] Sutherland MT, Stein EA. Functional neurocircuits and neuroimaging biomarkers of tobacco use disorder. Trends Mol Med. (2018) 24(2):129–43. doi: 10.1016/j.molmed.2017.12.002 29398401 PMC5928775

[12] Berridge KC, Robinson TE. Parsing reward. Trends Neurosci. (2003) 26(9):507–13. doi: 10.1016/S0166-2236(03)00233-9 12948663

[13] Berridge KC, Robinson TE. Liking, wanting, and the incentive-sensitization theory of addiction. Am Psychol. (2016) 71(8):670–9. doi: 10.1037/amp0000059 27977239 PMC5171207

[14] Salamone JD, Correa M, Farrar A, Mingote SM. Effort-related functions of nucleus accumbens dopamine and associated forebrain circuits. Psychopharmacology. (2007) 191:461–82. doi: 10.1007/s00213-006-0668-9 17225164

[15] Schultz W, Dayan P, Montague PR. A neural substrate of prediction and reward. Science. (1997) 275(5306):1593–9. doi: 10.1126/science.275.5306.1593 9054347

[16] O’Doherty JP, Dayan P, Friston K, Critchley H, Dolan RJ. Temporal difference models and reward-related learning in the human brain. Neuron. (2003) 38(2):329–37. doi: 10.1016/S0896-6273(03)00169-7 12718865

[17] Pessiglione M, Seymour B, Flandin G, Dolan RJ, Frith CD. Dopamine-dependent prediction errors underpin reward-seeking behaviour in humans. Nature. (2006) 442(7106):1042–5. doi: 10.1038/nature05051 16929307 PMC2636869

[18] Haber SN. Corticostriatal circuitry. Dialogues Clin Neurosci. (2016) 18(1):7–21. doi: 10.31887/DCNS.2016.18.1/shaber 27069376 PMC4826773

[19] Haber SN, Knutson B. The reward circuit: linking primate anatomy and human imaging. Neuropsychopharmacology. (2010) 35(1):4–26. doi: 10.1038/npp.2009.129 19812543 PMC3055449

[20] Balleine BW, O’Doherty PJ. Human and rodent homologies in action control: corticostriatal determinants of goal-directed and habitual action. Neuropsychopharmacology. (2010) 35(1):48–69. doi: 10.1038/npp.2009.131 19776734 PMC3055420

[21] Grill F, Guitart-Masip M, Johansson J, Stiernman L, Axelsson J, Nyberg L, Dopamine release in human associative striatum during reversal learning. Nat Commun. (2024) 15(1):59. doi: 10.1038/s41467-023-44358-w 38167691 PMC10762220

[22] O’Doherty PJ. Reward representations and reward-related learning in the human brain: insights from neuroimaging. Curr Opin Neurobiol. (2004) 14(6):769–76. doi: 10.1016/j.conb.2004.10.016 15582382

[23] Knutson B, Adams CM, Fong GW, Hommer D. Anticipation of increasing monetary reward selectively recruits nucleus accumbens. J Neurosci. (2001) 21(16): RC159. doi: 10.1523/JNEUROSCI.21-16-j0002.2001 11459880 PMC6763187

[24] Volkow ND, Morales M. The brain on drugs: from reward to addiction. Cell. (2015) 162(4):712–25. doi: 10.1016/j.cell.2015.07.046 26276628

[25] Hill-Bowen LD, Riedel MC, Poudel R, Salo T, Flannery JS, Camilleri JA, The cue-reactivity paradigm: an ensemble of networks driving attention and cognition when viewing drug and natural reward-related stimuli. Neurosci Biobehav Rev. (2021) 130:201–13. doi: 10.1016/j.neubiorev.2021.08.010 34400176 PMC8511211

[26] Balodis IM, Potenza MN. Anticipatory reward processing in addicted populations: a focus on the monetary incentive delay task. Biol Psychiatry. (2015) 77(5):434–44. doi: 10.1016/j.biopsych.2014.08.020 25481621 PMC4315733

[27] Bart CP, Nusslock R, Ng TH, Titone MK, Carroll AL, Damme KS, Decreased reward-related brain function prospectively predicts increased substance use. J Abnorm Psychol. (2021) 130(8):886. doi: 10.1037/abn0000711 34843292 PMC8634780

[28] Knutson B, Wimmer GE, Kuhnen CM, Winkielman P. Nucleus accumbens activation mediates the influence of reward cues on financial risk taking. Neuroreport. (2008) 19(5):509–13. doi: 10.1097/WNR.0b013e3282f85c01 18388729

[29] Cope LM, Martz ME, Hardee JE, Zucker RA, Heitzeg MM. Reward activation in childhood predicts adolescent substance use initiation in a high-risk sample. Drug Alcohol Depend. (2019) 194:318–25. doi: 10.1016/j.drugalcdep.2018.11.003 30471583 PMC6540995

[30] Beck A, Schlagenhauf F, Wustenberg T, Hein J, Kienast T, Kahnt T, Ventral striatal activation during reward anticipation correlates with impulsivity in alcoholics. Biol Psychiatry. (2009) 66(8):734–42. doi: 10.1016/j.biopsych.2009.04.035 19560123

[31] Robinson TE, Berridge KC. Review. The incentive sensitization theory of addiction: some current issues. Philos Trans R Soc Lond B Biol Sci. (2008) 363(1507):3137–46. doi: 10.1098/rstb.2008.0093 18640920 PMC2607325

[32] Goldstein RZ, Volkow ND. Dysfunction of the prefrontal cortex in addiction: neuroimaging findings and clinical implications. Nat Rev Neurosci. (2011) 12(11):652–69. doi: 10.1038/nrn3119 22011681 PMC3462342

[33] Büchel C, Peters J, Banaschewski T, Bokde AL, Bromberg U, Conrod PJ, Blunted ventral striatal responses to anticipated rewards foreshadow problematic drug use in novelty-seeking adolescents. Nat Commun. (2017) 8(1):14140. doi: 10.1038/ncomms14140 28221370 PMC5321762

[34] Swartz JR, Weissman DG, Ferrer E, Beard SJ, Fassbender C, Robins RW, Reward-Related brain activity prospectively predicts increases in alcohol use in adolescents. J Am Acad Child Adolesc Psychiatry. (2020) 59(3):391–400. doi: 10.1016/j.jaac.2019.05.022 31173884 PMC6891148

[35] Shulman EP, Smith AR, Silva K, Icenogle G, Duell N, Chein J, The dual systems model: review, reappraisal, and reaffirmation. Dev Cogn Neurosci. (2016) 17:103–17. doi: 10.1016/j.dcn.2015.12.010 26774291 PMC6990093

[36] Steinberg L. A dual systems model of adolescent risk-taking. Dev Psychobiol. (2010) 52(3):216–24. doi: 10.1002/dev.20445 20213754

[37] Wahlstrom D, Collins P, White T, Luciana M. Developmental changes in dopamine neurotransmission in adolescence: behavioral implications and issues in assessment. Brain Cogn. (2010) 72(1):146–59. doi: 10.1016/j.bandc.2009.10.013 19944514 PMC2815132

[38] Teicher MH, Andersen SL, Hostetter JC Jr. Evidence for dopamine receptor pruning between adolescence and adulthood in striatum but not nucleus accumbens. Brain Res Dev Brain Res. (1995) 89(2):167–72. doi: 10.1016/0165-3806(95)00109-Q 8612321

[39] Kuo HY, Yang YH, Chen SY, Kuo TH, Lin WT, Liu FC. Differential development of dendritic spines in striatal projection neurons of direct and indirect pathways in the caudoputamen and nucleus accumbens. eNeuro. (2023) 10(6):20. doi: 10.1523/ENEURO.0366-22.2023 PMC10270317 37253589

[40] van Duijvenvoorde ACK, van Hoorn J, Blankenstein NE. Risks and rewards in adolescent decision-making. Curr Opin Psychol. (2022) 48:101457. doi: 10.1016/j.copsyc.2022.101457 36088823

[41] Laursen B, Veenstra R. Toward understanding the functions of peer influence: a summary and synthesis of recent empirical research. J Res Adolesc. (2021) 31(4):889–907. doi: 10.1111/jora.12606 34820944 PMC8630732

[42] Nussenbaum K, Martin RE, Maulhardt S, Yang YJ, Bizzell-Hatcher G, Bhatt NS, Novelty and uncertainty differentially drive exploration across development. Elife. (2023):12. doi: 10.7554/eLife.84260 PMC10431916 37585251

[43] Bjork JM, Knutson B, Fong GW, Caggiano DM, Bennett SM, Hommer DW. Incentive-elicited brain activation in adolescents: similarities and differences from young adults. J Neurosci. (2004) 24(8):1793–802. doi: 10.1523/JNEUROSCI.4862-03.2004 14985419 PMC6730402

[44] Forbes EE, Hariri AR, Martin SL, Silk JS, Moyles DL, Fisher PM, Altered striatal activation predicting real-world positive affect in adolescent major depressive disorder. Am J Psychiatry. (2009) 166(1):64–73. doi: 10.1176/appi.ajp.2008.07081336 19047324 PMC2701209

[45] Bjork JM, Smith AR, Chen G, Hommer DW. Adolescents, adults and rewards: comparing motivational neurocircuitry recruitment using fMRI. PLoS One. (2010) 5(7):e11440. doi: 10.1371/journal.pone.0011440 20625430 PMC2897849

[46] Urosevic S, Collins P, Muetzel R, Schissel A, Lim KO, Luciana M. Effects of reward sensitivity and regional brain volumes on substance use initiation in adolescence. Soc Cogn Affect Neurosci. (2015) 10(1):106–13. doi: 10.1093/scan/nsu022 24526186 PMC4471534

[47] Tricomi EM, Delgado MR, Fiez JA. Modulation of caudate activity by action contingency. Neuron. (2004) 41(2):281–92. doi: 10.1016/S0896-6273(03)00848-1 14741108

[48] Guitart-Masip M, Huys QJ, Fuentemilla L, Dayan P, Duzel E, Dolan RJ. Go and no-go learning in reward and punishment: interactions between affect and effect. Neuroimage. (2012) 62(1):154–66. doi: 10.1016/j.neuroimage.2012.04.024 22548809 PMC3387384

[49] Whiteside SP, Lynam DR. The five factor model and impulsivity: using a structural model of personality to understand impulsivity. Pers Individ Dif. (2001) 30(4):669–89. doi: 10.1016/S0191-8869(00)00064-7

[50] Tarter RE, Kirisci L, Mezzich A, Cornelius JR, Pajer K, Vanyukov M, Neurobehavioral disinhibition in childhood predicts early age at onset of substance use disorder. Am J Psychiatry. (2003) 160(6):1078–85. doi: 10.1176/appi.ajp.160.6.1078 12777265

[51] Eisenberg N, Valiente C, Spinrad TL, Liew J, Zhou Q, Losoya SH, Longitudinal relations of children’s effortful control, impulsivity, and negative emotionality to their externalizing, internalizing, and co-occurring behavior problems. Dev Psychol. (2009) 45(4):988–1008. doi: 10.1037/a0016213 19586175 PMC2775424

[52] Castellanos-Ryan N, Parent S, Vitaro F, Tremblay RE, Seguin JR. Pubertal development, personality, and substance use: a 10-year longitudinal study from childhood to adolescence. J Abnorm Psychol. (2013) 122(3):782–96. doi: 10.1037/a0033133 24016016 PMC3812123

[53] Khurana A, Romer D, Betancourt LM, Brodsky NL, Giannetta JM, Hurt H. Experimentation versus progression in adolescent drug use: a test of an emerging neurobehavioral imbalance model. Dev Psychopathol. (2015) 27(3):901–13. doi: 10.1017/S0954579414000765 25154377 PMC4890960

[54] Beauchaine TP, Hinshaw SP, Pang KL. Comorbidity of attention-deficit/hyperactivity disorder and early-onset conduct disorder: biological, environmental, and developmental mechanisms. Clin Psychol Sci Pract. (2010) 17(4):327. doi: 10.1111/j.1468-2850.2010.01224.x

[55] Martel MM, Levinson CA, Lee CA, Smith TE. Impulsivity symptoms as core to the developmental externalizing spectrum. J Abnorm Child Psychol. (2017) 45:83–90. doi: 10.1007/s10802-016-0148-6 27017822 PMC5040618

[56] Carlson SR, Pritchard AA, Dominelli RM. Externalizing behavior, the UPPS-P impulsive behavior scale and reward and punishment sensitivity. Pers Individ Dif. (2013) 54(2):202–7. doi: 10.1016/j.paid.2012.08.039

[57] King SM, Iacono WG, McGue M. Childhood externalizing and internalizing psychopathology in the prediction of early substance use. Addiction. (2004) 99(12):1548–59. doi: 10.1111/j.1360-0443.2004.00893.x 15585046

[58] Littlefield AK, Sher KJ, Steinley D. Developmental trajectories of impulsivity and their association with alcohol use and related outcomes during emerging and young adulthood I. Alcohol Clin Exp Res. (2010) 34(8):1409–16. doi: 10.1111/j.1530-0277.2010.01224.x 20528822 PMC4260532

[59] Quinn PD, Harden KP. Differential changes in impulsivity and sensation seeking and the escalation of substance use from adolescence to early adulthood. Dev Psychopathol. (2013) 25(1):223–39. doi: 10.1017/S0954579412000284 22824055 PMC3967723

[60] Audrain-McGovern J, Rodriguez D, Epstein LH, Cuevas J, Rodgers K, Wileyto EP. Does delay discounting play an etiological role in smoking or is it a consequence of smoking? Drug Alcohol Depend. (2009) 103(3):99–106. doi: 10.1016/j.drugalcdep.2008.12.019 19443136 PMC2743449

[61] Galvan A, Hare TA, Parra CE, Penn J, Voss H, Glover G, Earlier development of the accumbens relative to orbitofrontal cortex might underlie risk-taking behavior in adolescents. J Neurosci. (2006) 26(25):6885–92. doi: 10.1523/JNEUROSCI.1062-06.2006 16793895 PMC6673830

[62] Plichta MM, Scheres A. Ventral-striatal responsiveness during reward anticipation in ADHD and its relation to trait impulsivity in the healthy population: a meta-analytic review of the fMRI literature. Neurosci Biobehav Rev. (2014) 38:125–34. doi: 10.1016/j.neubiorev.2013.07.012 23928090 PMC3989497

[63] Dalley JW, Fryer TD, Brichard L, Robinson ES, Theobald DE, Laane K, Nucleus accumbens D2/3 receptors predict trait impulsivity and cocaine reinforcement. Science. (2007) 315(5816):1267–70. doi: 10.1126/science.1137073 17332411 PMC1892797

[64] Buckholtz JW, Treadway MT, Cowan RL, Woodward ND, Li R, Ansari MS, Dopaminergic network differences in human impulsivity. Science. (2010) 329(5991):532. doi: 10.1126/science.1185778 20671181 PMC3161413

[65] Blum K, Braverman ER, Holder JM, Lubar JF, Monastra VJ, Miller D, The reward deficiency syndrome: a biogenetic model for the diagnosis and treatment of impulsive, addictive and compulsive behaviors. J Psychoact Drugs. (2000) 32(sup1):1–112. doi: 10.1080/02791072.2000.10736099 11280926

[66] Colder CR, Scalco M, Trucco EM, Read JP, Lengua LJ, Wieczorek WF, Prospective associations of internalizing and externalizing problems and their co-occurrence with early adolescent substance use. J Abnorm Child Psychol. (2013) 41:667–77. doi: 10.1007/s10802-012-9701-0 23242624 PMC3640685

[67] Heradstveit O, Skogen JC, Boe T, Hetland J, Pedersen MU, Hysing M. Prospective associations between childhood externalising and internalising problems and adolescent alcohol and drug use: the Bergen child study. Nordisk Alkohol Nark. (2018) 35(5):357–71. doi: 10.1177/1455072518789852 32934538 PMC7434147

[68] Valente J, Pietrobom T, Mihic J, Caetano S, Mari J, Sanchez ZM. Externalizing and internalizing problems as predictors of alcohol-related harm and binge drinking in early adolescence: the role of gender. J Affect Disord. (2023) 327:167–74. doi: 10.1016/j.jad.2023.01.004 36623566

[69] Windle M, Windle RC. Early onset problem behaviors and alcohol, tobacco, and other substance use disorders in young adulthood. Drug Alcohol Depend. (2012) 121(1–2):152–8. doi: 10.1016/j.drugalcdep.2011.08.024 21925804 PMC3247660

[70] Miettunen J, Murray GK, Jones PB, Maki P, Ebeling H, Taanila A, Longitudinal associations between childhood and adulthood externalizing and internalizing psychopathology and adolescent substance use. Psychol Med. (2014) 44(8):1727–38. doi: 10.1017/S0033291713002328 24028974

[71] Hawes SW, Waller R, Byrd AL, Bjork JM, Dick AS, Sutherland MT, Reward processing in children with disruptive behavior disorders and callous-unemotional traits in the ABCD study. Am J Psychiatry. (2021) 178(4):333–42. doi: 10.1176/appi.ajp.2020.19101092 32731811 PMC7855017

[72] Rodriguez-Thompson AM, Meyer KM, Davidow JY, Van Dijk KRA, Santillana RM, Snyder J, Examining cognitive control and reward interactions in adolescent externalizing symptoms. Dev Cogn Neurosci. (2020) 45:100813. doi: 10.1016/j.dcn.2020.100813 33040971 PMC7387777

[73] Elder J, Brieant A, Lauharatanahirun N, King-Casas B, Kim-Spoon J. Insular risk processing predicts alcohol use via externalizing pathway in male adolescents. J Stud Alcohol Drugs. (2019) 80(6):602–13. doi: 10.15288/jsad.2019.80.602 31790350 PMC6900996

[74] Bjork JM. The ups and downs of relating nondrug reward activation to substance use risk in adolescents. Curr Addict Rep. (2020) 7(3):421–9. doi: 10.1007/s40429-020-00327-7 33585160 PMC7880229

[75] Heitzeg MM, Villafuerte S, Weiland BJ, Enoch MA, Burmeister M, Zubieta JK, Effect of GABRA2 genotype on development of incentive-motivation circuitry in a sample enriched for alcoholism risk. Neuropsychopharmacology. (2014) 39(13):3077–86. doi: 10.1038/npp.2014.161 24975023 PMC4229579

[76] Heitzeg MM, Cope LM, Martz ME, Hardee JE. Neuroimaging risk markers for substance abuse: recent findings on inhibitory control and reward system functioning. Curr Addict Rep. (2015) 2:91–103. doi: 10.1007/s40429-015-0048-9 26236575 PMC4520315

[77] Stice E, Yokum S, Burger KS. Elevated reward region responsivity predicts future substance use onset but not overweight/obesity onset. Biol Psychiatry. (2013) 73(9):869–76. doi: 10.1016/j.biopsych.2012.11.019 23312561 PMC3774523

[78] Peters J, Bromberg U, Schneider S, Brassen S, Menz M, Banaschewski T, Lower ventral striatal activation during reward anticipation in adolescent smokers. Am J Psychiatry. (2011) 168(5):540–9. doi: 10.1176/appi.ajp.2010.10071024 21362742

[79] Martz ME, Hardee JE, Cope LM, McCurry KL, Soules M, Zucker RA, Nucleus Accumbens response to reward among children with a family history of alcohol use problems: convergent findings from the ABCD study((R)) and Michigan longitudinal study. Brain Sci. (2022) 12(7):8. doi: 10.3390/brainsci12070913 PMC9320357 35884720

[80] Martz ME, Trucco EM, Cope LM, Hardee JE, Jester JM, Zucker RA, Association of marijuana use with blunted nucleus accumbens response to reward anticipation. JAMA Psychiatry. (2016) 73(8):838–44. doi: 10.1001/jamapsychiatry.2016.1161 27384542 PMC4972653

[81] Scheres A, Milham MP, Knutson B, Castellanos FX. Ventral striatal hyporesponsiveness during reward anticipation in attention-deficit/hyperactivity disorder. Biol Psychiatry. (2007) 61(5):720–4. doi: 10.1016/j.biopsych.2006.04.042 16950228

[82] Mumford JA, Demidenko MI, Bjork JM, Chaarani B, Feczko EJ, Garavan HP, Unintended bias in the pursuit of collinearity solutions in fMRI analysis. Imaging Neurosci (Camb). (2025) 3. doi: 10.1162/IMAG.a.958 PMC12550278 41143079

[83] Cristello JV, Sutherland MT, Trucco EM. A preliminary validation of the adolescent e-cigarette consequences questionnaire. Drug Alcohol Depend. (2020) 213:108118. doi: 10.1016/j.drugalcdep.2020.108118 32559666 PMC7371533

[84] Hartmann SA, Hayes T, Sutherland MT, Trucco EM. Risk factors for early use of e-cigarettes and alcohol: dimensions and profiles of temperament. Dev Psychopathol. (2021) 35(2):481–93. doi: 10.1017/S0954579421001565 34924096 PMC9207150

[85] Sutherland BD, Perez PMV, Crooks KE, Flannery JS, Hill-Bowen LD, Riedel MC, The association of amygdala-insula functional connectivity and adolescent e-cigarette use via sleep problems and depressive symptoms. Addict Behav. (2022) 135:107458. doi: 10.1016/j.addbeh.2022.107458 35998541 PMC9730909

[86] Sutherland BD, Sutherland MT, Trucco EM. Electronic cigarette use intentions mediate the association between low self-control and future use by internalizing symptoms. Subst Use Misuse. (2022) 57(12):1797–807. doi: 10.1080/10826084.2022.2115848 36041007 PMC9560985

[87] Trucco EM, Cristello JV, Sutherland MT. Do parents still matter? The impact of parents and peers on adolescent electronic cigarette use. J Adolesc Health. (2021) 68(4):780–6. doi: 10.1016/j.jadohealth.2020.12.002 33431246 PMC8012253

[88] Sutherland BD, Hill-Bowen LD, Trucco EM, Laird AR, Sutherland MT. Working memory-related brain activations and deactivations linked with adolescent substance use via alexithymia. Dev Cogn Neurosci. (2025) 76:101634. doi: 10.1016/j.dcn.2025.101634 41101078 PMC12552979

[89] Cyders MA, Littlefield AK, Coffey S, Karyadi KA. Examination of a short English version of the UPPS-P impulsive behavior scale. Addict Behav. (2014) 39(9):1372–6. doi: 10.1016/j.addbeh.2014.02.013 24636739 PMC4055534

[90] Watts AL, Smith GT, Barch DM, Sher KJ. Factor structure, measurement and structural invariance, and external validity of an abbreviated youth version of the UPPS-P impulsive behavior scale. Psychol Assess. (2020) 32(4):336–47. doi: 10.1037/pas0000791 31841018 PMC7093245

[91] Whiteside SP, Lynam DR, Miller JD, Reynolds SK. Validation of the UPPS impulsive behaviour scale: a four-factor model of impulsivity. Eur J Pers. (2005) 19(7):559–74. doi: 10.1002/per.556

[92] Gilman JM, Kaur J, Tervo-Clemmens B, Potter K, Sanzo BT, Schuster RM, Associations between behavioral and self-reported impulsivity, brain structure, and genetic influences in middle childhood. Dev Cogn Neurosci. (2024) 67:101389. doi: 10.1016/j.dcn.2024.101389 38749217 PMC11112269

[93] Achenbach TM. The Achenbach System of Empirically Based Assessment (ASEBA): Development, Findings, Theory, and Applications. Burlington Vermont: University of Vermont, Research Center for Children, Youth, & Families (2009).

[94] Trucco EM, Colder CR, Wieczorek WF, Lengua LJ, Hawk LW Jr. Early adolescent alcohol use in context: how neighborhoods, parents, and peers impact youth. Dev Psychopathol. (2014) 26(2):425–36. doi: 10.1017/S0954579414000042 24621660 PMC4073105

[95] Hyland A, Ambrose BK, Conway KP, Borek N, Lambert E, Carusi C, Design and methods of the population assessment of tobacco and health (PATH) study. Tob Control. (2016) 26:371–8. doi: 10.1136/tobaccocontrol-2016-052934 27507901 PMC5299069

[96] Rose EJ, Ross TJ, Salmeron BJ, Lee M, Shakleya DM, Huestis MA, Acute nicotine differentially impacts anticipatory valence-and magnitude-related striatal activity. Biol Psychiatry. (2013) 73(3):280–8. doi: 10.1016/j.biopsych.2012.06.034 22939991 PMC9361221

[97] Fedota JR, Sutherland MT, Salmeron BJ, Ross TJ, Hong LE, Stein EA. Reward anticipation is differentially modulated by varenicline and nicotine in smokers. Neuropsychopharmacology. (2015) 40(8):2038–46. doi: 10.1038/npp.2015.54 25742873 PMC4839527

[98] Esteban O, Birman D, Schaer M, Koyejo OO, Poldrack RA, Gorgolewski KJ. MRIQC: advancing the automatic prediction of image quality in MRI from unseen sites. PLoS One. (2017) 12(9):21. doi: 10.1371/journal.pone.0184661 PMC5612458 28945803

[99] Esteban O, Blair R, Markiewicz CJ, Berleant SL, Moodie C, Ma F, FMRIPrep.: Software. Zenodo (2018).

[100] Cox RW, Hyde JS. Software tools for analysis and visualization of fMRI data. NMR Biomed. (1997) 10:171–8. doi: 10.1002/(SICI)1099-1492(199706/08)10:4/5fl171::AID-NBM453>3.0.CO;2-L 9430344

[101] Esteban O, Markiewicz CJ, Blair RW, Moodie CA, Isik AI, Erramuzpe A, fMRIPrep: a robust preprocessing pipeline for functional MRI. Nat Methods. 2019;16(1):111–6. doi: 10.1038/s41592-018-0235-4 30532080 PMC6319393

[102] Nichols TE, Das S, Eickhoff SB, Evans AC, Glatard T, Hanke M, Best practices in data analysis and sharing in neuroimaging using MRI. Nat Neurosci. (2017) 20(3):299–303. doi: 10.1038/nn.4500 28230846 PMC5685169

[103] Yarkoni T, Poldrack RA, Nichols TE, Van Essen DC, Wager TD. Large-scale automated synthesis of human functional neuroimaging data. Nat Methods. (2011) 8(8):665–70. doi: 10.1038/nmeth.1635 21706013 PMC3146590

[104] Chen G, Saad ZS, Britton JC, Pine DS, Cox RW. Linear mixed-effects modeling approach to FMRI group analysis. Neuroimage. (2013) 73:176–90. doi: 10.1016/j.neuroimage.2013.01.047 23376789 PMC3638840

[105] RStudio Team U. RStudio: Integrated Development for R. (2015) 42:14. Boston, MA: RStudio, Inc. Available online at: http://www.rstudio.com (Accessed January 22, 2026).

[106] Hayes AF. Introduction to Mediation, Moderation, and Conditional Process Analysis: A Regression-Based approach. New York: Guildford Press (2017).

[107] Hurley DB, Kwon P. Results of a study to increase savoring the moment: differential impact on positive and negative outcomes. J Happiness Stud. (2012) 13(4):579–88. doi: 10.1007/s10902-011-9280-8

[108] Knutson B, Westdorp A, Kaiser E, Hommer D. FMRI visualization of brain activity during a monetary incentive delay task. Neuroimage. (2000) 12(1):20–7. doi: 10.1006/nimg.2000.0593 10875899

[109] Kim H. Involvement of the dorsal and ventral attention networks in oddball stimulus processing: a meta-analysis. Hum Brain Mapp. (2014) 35(5):2265–84. doi: 10.1002/hbm.22326 23900833 PMC6868981

[110] Harsay HA, Spaan M, Wijnen JG, Ridderinkhof KR. Error awareness and salience processing in the oddball task: shared neural mechanisms. Front Hum Neurosci. (2012) 6:246. doi: 10.3389/fnhum.2012.00246 22969714 PMC3427876

[111] Menon V, Uddin LQ. Saliency, switching, attention and control: a network model of insula function. Brain Structure and Function. (2010) 214:655–67. doi: 10.1007/s00429-010-0262-0 20512370 PMC2899886

[112] Knutson B, Fong GW, Bennett SM, Adams CM, Hommer D. A region of mesial prefrontal cortex tracks monetarily rewarding outcomes: characterization with rapid event-related fMRI. Neuroimage. (2003) 18(2):263–72. doi: 10.1016/S1053-8119(02)00057-5 12595181

[113] Miller EM, Shankar MU, Knutson B, McClure SM. Dissociating motivation from reward in human striatal activity. J Cogn Neurosci. (2014) 26(5):1075–84. doi: 10.1162/jocn_a_00535 24345173 PMC5808846

[114] Kurniawan IT, Seymour B, Talmi D, Yoshida W, Chater N, Dolan RJ. Choosing to make an effort: the role of striatum in signaling physical effort of a chosen action. J Neurophysiol. (2010) 104(1):313–21. doi: 10.1152/jn.00027.2010 20463204 PMC2904211

[115] Volkow ND, Koob GF, McLellan AT. Neurobiologic advances from the brain disease model of addiction. N Engl J Med. (2016) 374(4):363–71. doi: 10.1056/NEJMra1511480 26816013 PMC6135257

[116] Koob GF, Volkow ND. Neurobiology of addiction: a neurocircuitry analysis. Lancet Psychiatry. (2016) 3(8):760–73. doi: 10.1016/S2215-0366(16)00104-8 27475769 PMC6135092

[117] Galvan A. Adolescent development of the reward system. Front Hum Neurosci. (2010) 4:6. doi: 10.3389/neuro.09.006.2010 20179786 PMC2826184

[118] Kim B, Im HI. The role of the dorsal striatum in choice impulsivity. Ann N Y Acad Sci. (2019) 1451(1):92–111. doi: 10.1111/nyas.13961 30277562

[119] Smith CT, Juan S, Dang MD, Katz LC, Perkins DT, Burgess SF, Ventral striatal dopamine transporter availability is associated with lower trait motor impulsivity in healthy adults. Transl Psychiatry. (2018) 8(1):269. doi: 10.1038/s41398-018-0328-y 30531858 PMC6286354

[120] Dalley JW, Roiser J. Dopamine, serotonin and impulsivity. Neuroscience. (2012) 215:42–58. doi: 10.1016/j.neuroscience.2012.03.065 22542672

[121] London ED. Human brain imaging links dopaminergic systems to impulsivity. In: de Wit H, Jentsch JD, editors. Recent Advances in Research on Impulsivity and Impulsive Behaviors. Current Topics in Behavioral Neurosciences, Vol. 47. Cham: Springer (2020). doi: 10.1007/7854_2019_125 31907881

[122] Weiland BJ, Heitzeg MM, Zald D, Cummiford C, Love T, Zucker RA, Relationship between impulsivity, prefrontal anticipatory activation, and striatal dopamine release during rewarded task performance. Psychiatry Res Neuroimaging. (2014) 223(3):244–52. doi: 10.1016/j.pscychresns.2014.05.015 PMC4136473 24969539

[123] Schott BH, Minuzzi L, Krebs RM, Elmenhorst D, Lang M, Winz OH, Mesolimbic functional magnetic resonance imaging activations during reward anticipation correlate with reward-related ventral striatal dopamine release. J Neurosci. (2008) 28(52):14311–9. doi: 10.1523/JNEUROSCI.2058-08.2008 19109512 PMC6671462

[124] Cools R, D’Esposito M. Inverted-U-shaped dopamine actions on human working memory and cognitive control. Biol Psychiatry. (2011) 69(12):e113–25. doi: 10.1016/j.biopsych.2011.03.028 21531388 PMC3111448

[125] Robbins TW, Arnsten AF. The neuropsychopharmacology of fronto-executive function: monoaminergic modulation. Annu Rev Neurosci. (2009) 32:267–87. doi: 10.1146/annurev.neuro.051508.135535 19555290 PMC2863127

[126] van den Bosch R, Lambregts B, Maatta J, Hofmans L, Papadopetraki D, Westbrook A, Striatal dopamine dissociates methylphenidate effects on value-based versus surprise-based reversal learning. Nat Commun. (2022) 13(1):4962. doi: 10.1038/s41467-022-32679-1 36002446 PMC9402573

[127] Volkow ND, Wang GJ, Tomasi D, Kollins SH, Wigal TL, Newcorn JH, Methylphenidate-elicited dopamine increases in ventral striatum are associated with long-term symptom improvement in adults with attention deficit hyperactivity disorder. J Neurosci. (2012) 32(3):841–9. doi: 10.1523/JNEUROSCI.4461-11.2012 22262882 PMC3350870

[128] Spencer TJ, Brown A, Seidman LJ, Valera EM, Makris N, Lomedico A, Effect of psychostimulants on brain structure and function in ADHD: a qualitative literature review of magnetic resonance imaging-based neuroimaging studies. J Clin Psychiatry. (2013) 74(9):902–17. doi: 10.4088/JCP.12r08287 24107764 PMC3801446

[129] Arnsten AF. The emerging neurobiology of attention deficit hyperactivity disorder: the key role of the prefrontal association Cortex. J Pediatr. (2009) 154(5):1–S43. doi: 10.1016/j.jpeds.2009.01.018 20596295 PMC2894421

[130] Di Chiara G, Imperato A. Drugs abused by humans preferentially increase synaptic dopamine concentrations in the mesolimbic system of freely moving rats. Proc Natl Acad Sci U S A. (1988) 85(14):5274–8. doi: 10.1073/pnas.85.14.5274 2899326 PMC281732

[131] Mansvelder HD, Keath JR, McGehee DS. Synaptic mechanisms underlie nicotine-induced excitability of brain reward areas. Neuron. (2002) 33(6):905–19. doi: 10.1016/S0896-6273(02)00625-6 11906697

[132] Brody AL, Olmstead RE, London ED, Farahi J, Meyer JH, Grossman P, Smoking-induced ventral striatum dopamine release. Am J Psychiatry. (2004) 161(7):1211–8. doi: 10.1176/appi.ajp.161.7.1211 15229053

[133] Bloomfield MA, Ashok AH, Volkow ND, Howes OD. The effects of Delta(9)-tetrahydrocannabinol on the dopamine system. Nature. (2016) 539(7629):369–77. doi: 10.1038/nature20153 27853201 PMC5123717

[134] Cheer JF, Wassum KM, Sombers LA, Heien ML, Ariansen JL, Aragona BJ, Phasic dopamine release evoked by abused substances requires cannabinoid receptor activation. J Neurosci. (2007) 27(4):791–5. doi: 10.1523/JNEUROSCI.4152-06.2007 17251418 PMC6672925

[135] Tiwari RK, Sharma V, Pandey RK, Shukla SS. Nicotine addiction: neurobiology and mechanism. J Pharmacopuncture. (2020) 23(1):1. doi: 10.3831/KPI.2020.23.001 32322429 PMC7163392

[136] Weiss F, Porrino LJ. Behavioral neurobiology of alcohol addiction: recent advances and challenges. J Neurosci. (2002) 22(9):3332–7. doi: 10.1523/JNEUROSCI.22-09-03332.2002 11978808 PMC6758393

[137] Koistinen M, Tuomainen P, Hyytiä P, Kiianmaa K. Naltrexone suppresses ethanol intake in 6-hydroxydopamine–treated rats. Alcohol Clin Exp Res. (2001) 25(11):1605–12. doi: 10.1111/j.1530-0277.2001.tb02167.x 11707635

[138] Engleman EA, McBride WJ, Wilber AA, Shaikh SR, Eha RD, Lumeng L, Reverse microdialysis of a dopamine uptake inhibitor in the nucleus accumbens of alcohol-preferring rats: effects on dialysate dopamine levels and ethanol intake. Alcohol Clin Exp Res. (2000) 24(6):795–801. doi: 10.1097/00000374-200006000-00008 10888067

[139] Erdozain AM, Callado LF. Neurobiological alterations in alcohol addiction: a review. Adicciones. (2014) 26(4):360–70. doi: 10.20882/adicciones.40 25578004

[140] Zilverstand A, Huang AS, Alia-Klein N, Goldstein RZ. Neuroimaging impaired response inhibition and salience attribution in human drug addiction: a systematic review. Neuron. (2018) 98(5):886–903. doi: 10.1016/j.neuron.2018.03.048 29879391 PMC5995133

[141] Hussong AM, Jones DJ, Stein GL, Baucom DH, Boeding S. An internalizing pathway to alcohol use and disorder. Psychol Addict Behav. (2011) 25(3):390–404. doi: 10.1037/a0024519 21823762 PMC3178003

[142] Zaso MJ, Read JP, Colder CR. Coping-motivated escalations in adolescent alcohol problems following early adversity. Psychol Addict Behav. (2023) 37(2):331–40. doi: 10.1037/adb0000788 34618492 PMC8986882

[143] Colder CR, Lee YH, Frndak S, Read JP, Wieczorek WF. Internalizing symptoms and cannabis and alcohol use: between- and within-person risk pathways with coping motives. J Consult Clin Psychol. (2019) 87(7):629–44. doi: 10.1037/ccp0000413 31219294 PMC7140101

[144] Trucco EM. A review of psychosocial factors linked to adolescent substance use. Pharmacol Biochem Behav. (2020) 196:172969. doi: 10.1016/j.pbb.2020.172969 32565241 PMC7415605

[145] Trucco EM, Colder CR, Wieczorek WF. Vulnerability to peer influence: a moderated mediation study of early adolescent alcohol use initiation. Addict Behav. (2011) 36(7):729–36. doi: 10.1016/j.addbeh.2011.02.008 21420241 PMC3088763

[146] Harrison ME, Kanbur N, Canton K, Desai TS, Lim-Reinders S, Groulx C, Adolescents’ cannabis knowledge and risk perception: a systematic review. J Adolesc Health. (2024) 74(3):402–40. doi: 10.1016/j.jadohealth.2023.09.014 37966406

[147] Khodaee A, Reed A, Khodaee M. Electronic cigarette use (vaping) among adolescents: a narrative review of an emerging public health epidemic. Cureus. (2025) 17(8):e89422. doi: 10.7759/cureus.89422 40918883 PMC12410507

[148] Bryant FB, Veroff J. Savoring: A New Model of Positive Experience. Mahwah, NJ: Lawrence Erlbaum Associates (2007).

[149] Jose PE, Lim BT, Bryant FB. Does savoring increase happiness? A daily diary study. J Posit Psychol. (2012) 7(3):176–87. doi: 10.1080/17439760.2012.671345

[150] Quoidbach J, Berry EV, Hansenne M, Mikolajczak M. Positive emotion regulation and well-being: comparing the impact of eight savoring and dampening strategies. Pers Individ Dif. (2010) 49(5):368–73. doi: 10.1016/j.paid.2010.03.048

[151] Guldner S, Prignitz M, Nees F, Consortium IM-M. Mindfulness facets are differentially related with reward processing stages in striatum and alcohol use in adolescence. Prog Neuropsychopharmacol Biol Psychiatry. (2024) 135:111113. doi: 10.1016/j.pnpbp.2024.111113 39094927

[152] Young CB, Nusslock R. Positive mood enhances reward-related neural activity. Soc Cogn Affect Neurosci. (2016) 11(6):934–44. doi: 10.1093/scan/nsw012 26833919 PMC4884311

[153] Finan PH, Hunt C, Keaser ML, Smith K, Lerman S, Bingham CO, Effects of savoring meditation on positive emotions and pain-related brain function: a mechanistic randomized controlled trial in people with rheumatoid arthritis. J Pain. (2024) 25(7):104478. doi: 10.1016/j.jpain.2024.01.343 38244899

[154] Froeliger B, Mathew AR, McConnell PA, Eichberg C, Saladin ME, Carpenter MJ, Restructuring reward mechanisms in nicotine addiction: a pilot fMRI study of mindfulness-oriented recovery enhancement for cigarette smokers. Evid Based Complement Alternat Med. (2017) 2017:7018014 (10 pages). doi: 10.1155/2017/7018014 28373890 PMC5360937

[155] Garland EL, Atchley RM, Hanley AW, Zubieta JK, Froeliger B. Mindfulness-oriented recovery enhancement remediates hedonic dysregulation in opioid users: neural and affective evidence of target engagement. Sci Adv. (2019) 5(10):eaax1569. doi: 10.1126/sciadv.aax1569 PMC6795512 31663023

[156] Waldron HB, Turner CW. Evidence-based psychosocial treatments for adolescent substance abuse. J Clin Child Adolesc Psychol. (2008) 37(1):238–61. doi: 10.1080/15374410701820133 18444060

[157] Carney T, Myers B. Effectiveness of early interventions for substance-using adolescents: findings from a systematic review and meta-analysis. Subst Abuse Treat Prev Policy. (2012) 7:25. doi: 10.1186/1747-597X-7-25 22697269 PMC3538561

[158] Kober H, Mende-Siedlecki P, Kross EF, Weber J, Mischel W, Hart CL, Prefrontal-striatal pathway underlies cognitive regulation of craving. Proc Natl Acad Sci U S A. (2010) 107(33):14811–6. doi: 10.1073/pnas.1007779107 20679212 PMC2930456

[159] Seeley WW, Menon V, Schatzberg AF, Keller J, Glover GH, Kenna H, Dissociable intrinsic connectivity networks for salience processing and executive control. J Neurosci. (2007) 27(9):2349–56. doi: 10.1523/JNEUROSCI.5587-06.2007 17329432 PMC2680293

[160] Ullsperger M, Danielmeier C, Jocham G. Neurophysiology of performance monitoring and adaptive behavior. Physiol Rev. (2014) 94(1):35–79. doi: 10.1152/physrev.00041.2012 24382883

[161] Kim JJ, Perez VM, Gonzales NA, Thamrin H, Tein JY. Measurement and functional equivalence of a reduced version of the UPPS impulsivity scale among Hispanic, non-Hispanic black, and non-Hispanic white adolescents. Assessment. (2023) 30(6):1895–913. doi: 10.1177/10731911221129243 36254674 PMC10268942

[162] McLaughlin KA, Hilt LM, Nolen-Hoeksema S. Racial/ethnic differences in internalizing and externalizing symptoms in adolescents. J Abnorm Child Psychol. (2007) 35(5):801–16. doi: 10.1007/s10802-007-9128-1 17508278 PMC2881593

[163] Temourian AA, Ling PM, Do VV, Nguyen N. Racial and ethnic disparities in cannabis use among U.S. youth who use tobacco: findings from the population assessment of tobacco and health study. AJPM Focus. (2025) 4(5):100392. doi: 10.1016/j.focus.2025.100392 40988876 PMC12451367

[164] Goodwin RD, Silverman KD. Evolving disparities in Cannabis use among youth by demographics and tobacco and alcohol use in the U.S.: 2013–2021. Am J Prev Med. (2024) 66(6):1035–42. doi: 10.1016/j.amepre.2024.01.012 38272242

[165] Zheng S, Stewart SL, Keegan TH, Tong EK, Dove MS. The association between home language, self-identified Hispanic origin, and current e-cigarette use among Hispanic youth in the United States—national youth tobacco survey, 2022–2023. Prev Med Rep. (2025) 53:103065. doi: 10.1016/j.pmedr.2025.103065 40256406 PMC12008535

[166] Okine L, Unger JB. Substance use among Latinx youth: the roles of sociocultural influences, family factors, and childhood adversity. J Res Adolesc. (2024) 34(4):1562–72. doi: 10.1111/jora.13025 39400443 PMC11606254

[167] Johnston LD, Miech RA, Malley O, Bachman JGPM, Schulenberg JE, Patrick ME. Monitoring the Future National Survey Results on Drug Use 1975–2019: Overview, Key Findings on Adolescent Drug Use. Ann Arbor, MI: Institute for Social Research, University of Michigan (2019).

[168] Johnston LD, Miech RA, Malley O, Bachman JGPM, Schulenberg JE, Patrick ME. Monitoring the Future National Survey Results on Drug Use 1975–2021: Overview, Key Findings on Adolescent Drug Use. Ann Arbor, MI: Institute for Social Research, University of Michigan (2021).

[169] Brener ND, Billy JOG, Grady WR. Assessment of factors affecting the validity of self-reported health-risk behavior among adolescents: evidence from the scientific literature. J Adolesc Health. (2003) 33(6):436–57. doi: 10.1016/S1054-139X(03)00052-1 14642706

[170] Betka S, Pfeifer G, Garfinkel S, Prins H, Bond R, Sequeira H, How do self-assessment of alexithymia and sensitivity to bodily sensations relate to alcohol consumption? Alcohol Clin Exp Res. (2018) 42(1):81–8. doi: 10.1111/acer.13542 29094768

[171] Casey BJ, Cannonier T, Conley MI, Cohen AO, Barch DM, Heitzeg MM, The adolescent brain cognitive development (ABCD) study: imaging acquisition across 21 sites. Dev Cogn Neurosci. (2018) 32:43–54. doi: 10.1016/j.dcn.2018.03.001 29567376 PMC5999559

