# Supplementary material for: Blunted reward-related striatal activity and behavioral disinhibition as a pathway to adolescent cannabis and e-cigarette use
Source: Front Adolesc Med. Author manuscript; Available in PMC 2026 Jul 29. (PMC13411140; doi:10.3389/fradm.2026.1741884)
Supplement: Supplemental Material [file NIHMS2196548-supplement-Supplemental_Material.docx]

**SUPPLEMENTAL INFORMATION**

**Blunted reward-related striatal activity and behavioral disinhibition as a pathway to adolescent cannabis and e-cigarette use**

Patricio M. Viera Perez, Lauren D. Hill, Katharine E. Crooks, Benjelene D. Sutherland, Angela R. Laird, Elisa M. Trucco, and Matthew T. Sutherland

**SUPPLEMENTAL CONTENT**

**TEXT**

**MRI data preprocessing** 3

**Post-hoc sensitivity analyses** 5

**TABLES**

**Table S1.** Substance use metrics by data collection wave 6

**Table S2.** Descriptives and correlations across study variables 7

**Table S3.** Serial mediation outcomes linking brain, impulsivity, externalizing, and cannabis use 8

**Table S4.** Serial mediation outcomes linking brain, impulsivity, externalizing, and e-cigarette use 9

**Table S5.** Serial mediation outcomes linking brain, impulsivity, externalizing, and alcohol use 10

**Table S6.** Serial mediation outcomes linking brain, impulsivity, externalizing, and cannabis use controlling for multiple substances 11

**Table S7.** Serial mediation outcomes linking brain, impulsivity, externalizing, and e-cigarette use controlling for multiple substances 12

**Table S8.** Serial mediation outcomes linking brain, impulsivity, externalizing, and alcohol use controlling for multiple substances 13

**FIGURES**

**Figure S1.** MID task schematic 14

**Figure S2.** Small volume corrected (SVC) analysis mask 15

**Figure S3.** Serial mediation models linking right striatal (caudate) activity, impulsivity, externalizing, and future substance use 16

**SUPPLEMENTAL REFERENCES** 17

**MRI data** **preprocessing.**

MRI data were preprocessed using fMRIPrep 20.2.1 (1) (RRID:SCR_016216), based on Nipype 1.5.1 (2) (RRID:SCR_002502).

Anatomical images. T1-weighted (T1w) images were corrected for intensity non-uniformity with N4BiasFieldCorrection(3), distributed with ANTs 2.3.3 (RRID: SCR_004757) (4)and used as a T1w-reference throughout the workflow. The T1w-reference was then skull-stripped with a Nipype implementation of the *antsBrainExtraction.sh* workflow, using OASIS30ANTs as the target template. Brain tissue segmentation of cerebrospinal fluid (CSF), white-matter (WM) and gray-matter (GM) was performed on the brain-extracted T1w image using *FAST* (FSL 5.0.9, RRID: SCR_002823)(5). Brain surfaces were reconstructed using *recon-all* (FreeSurfer 6.0.1, RRID: SCR_001847)(6), and the brain mask estimated previously was refined with a custom variation of the method to reconcile ANTs-derived and FreeSurfer-derived segmentations of the cortical gray-matter of *Mindboggle* (RRID: SCR_002438)(7). Volume-based spatial normalization to standard space (MNI152NLin2009cAsym) was performed through nonlinear registration with *antsRegistration* (ANTs 2.3.3), using brain-extracted versions of both the T1w-reference and the T1w-template. The following template was selected for spatial normalization: ICBM 152 Nonlinear Asymmetrical template version 2009c (RRID: SCR_008796; TemplateFlow ID: MNI152Nlin2009cAsym)(8).

Functional images. For each of the 4 MID BOLD runs per subject, the following preprocessing was performed. First, a reference volume and its skull-stripped version were generated using a custom methodology of fMRIPrep. A B0-nonuniformity map (i.e., field map) was estimated based on two (or more) echo-planar imaging (EPI) references with opposing phase-encoding directions, using *3dQwarp* (AFNI 20160207)(9). Based on the estimated susceptibility distortion, a corrected EPI reference was calculated for a more accurate co-registration with the anatomical reference. The BOLD reference was then co-registered to the T1w-reference using *bbregister* (FreeSurfer) which implements boundary-based registration(10). Co-registration was configured with six degrees of freedom. Head-motion parameters with respect to the BOLD reference (transformation matrices, and six corresponding rotation and translation parameters) were estimated before any spatiotemporal filtering using *mcflirt* (FSL 5.0.9)(11). BOLD runs were slice-time corrected using AFNI’s *3dTshift* (20160207, RRID:SCR_005927)(9). The BOLD time-series (including slice-timing correction when applied) were resampled onto their original, native space by applying a single, composite transform to correct for head-motion and susceptibility distortions. These resampled BOLD time-series will be referred to as preprocessed BOLD images in original space or just preprocessed BOLD images. The BOLD time-series were resampled into standard space, generating a preprocessed BOLD run in MNI152Nlin2009cAsym space. First, a reference volume and its skull-stripped version were generated using a custom methodology of fMRIPrep. Several confounding time-series were calculated based on the preprocessed BOLD: framewise displacement (FD), DVARS, and three region-wise global signals. FD was computed using two formulations following Power and Jenkinson, absolute sum of relative motions (12) and relative root mean square displacement between affines(11). FD and DVARS were calculated for each functional run, both using their implementations in Nipype following the definitions byPower, Mitra (12).

Three global signals were extracted from within the CSF, WM, and whole-brain masks. Additionally, a set of physiological regressors were extracted to allow for component-based noise correction (CompCor)(13). Principal components were estimated after high-pass filtering the preprocessed BOLD time-series (using a discrete cosine filter with 128s cut-off) for the two CompCor variants: temporal (tCompCor) and anatomical (aCompCor). tCompCor components were then calculated from the top 2% variable voxels within the brain mask. For aCompCor, three probabilistic masks (CSF, WM, and combined CSF+WM) were generated in anatomical space. This implementation differs from that of Behzadi, Restom (13) in that instead of eroding the masks by 2 pixels on BOLD space, the aCompCor WM masks were subtracted from a mask of pixels that likely contain a volume fraction of GM. This mask was obtained by dilating a GM mask extracted from the FreeSurfer’s aseg segmentation and helped to ensure components were not extracted from voxels containing a minimal fraction of GM. Finally, these masks were resampled into BOLD space and binarized by thresholding at 0.99 (as in the original implementation). Components were also calculated separately within the WM and CSF masks. For each CompCor decomposition, the *k* components with the largest singular values were retained, such that the retained components’ time series were sufficient to explain 50% of the variance across the nuisance mask (CSF, WM, combined, or temporal). The remaining components were dropped from consideration. The head-motion estimates calculated in the correction step were also placed within the corresponding confounds file. The confounded time series derived from head motion estimates and global signals were expanded with the inclusion of temporal derivatives and quadratic terms for each(14). Frames that exceeded a threshold of 0.5-mm FD or 1.5 standardized DVARS were annotated as motion outliers. All resamplings were performed with a single interpolation step by composing all the pertinent transformations (i.e., head-motion transform matrices, susceptibility distortion correction when available, and co-registrations to anatomical and output spaces). Gridded (volumetric) resamplings were performed using *antsApplyTransforms* (ANTs), configured with Lanczos interpolation to minimize the smoothing effects of other kernels(15). Non-gridded (surface) resamplings were performed using *mri_vol2surf* (FreeSurfer).

**Post-hoc sensitivity analyses.**

We conducted *post-hoc* serial mediation analyses to assess possible alternative orderings. First, we assessed whether impulsivity (X) predicted future SU (Y) indirectly via striatal activity (M_1_) and externalizing (M_2_). In other words, we “swapped the order” of the X and M_1_, variables which were collected at the same time point. We observed that impulsivity was linked with striatal activity (estimate = -0.21, *p* = 0.02), however, striatal activity did not directly predict externalizing behaviors (estimate = 0.08, *p* = 0.32) or cannabis use (estimate = 0.08, *p* = 0.15). That is, we did not detect evidence of serial mediation (indirect effect = -0.003, CI [-0.01, 0.005]). Rather, there was support for simple mediation via externalizing behaviors (indirect effect = 0.06, CI [0.01, 0.14]), where more impulsivity predicted higher externalizing scores (estimate = 0.34, *p* < 0.001), which in turn predicted more cannabis use days (estimate = 0.17, *p* = 0.004). Given that impulsivity linked with both striatal activity and externalizing behaviors, we retained the original model ordering. Similarly, for e-cigarette use we did not detect evidence of serial mediation (indirect effect = -0.01, CI [-0.02, 0.01]) given that striatal activity did not directly predict externalizing behaviors (estimate = 0.07, *p* = 0.35). Second, we assessed whether striatal activity (X) predicted future externalizing behaviors (Y) indirectly via impulsivity (M_1_) and cannabis use (M_2_). In other words, we “swapped the order” of the M_2_ and Y, variables which were collected at the same time point. Again, we did not detect evidence of serial mediation (indirect effect = -0.002, CI [-0.02, 0.01]) as impulsivity (M_1_) did not directly predict cannabis use (M_2_, estimate = 0.03, *p* = 0.62) although cannabis use was linked with externalizing scores (estimate = 0.35, *p* = 0.004). Rather, there was support for simple mediation via impulsivity, where less striatal activity was linked with more impulsivity which in turn predicted more externalizing behaviors (indirect effect = -0.07, CI [-0.14, -0.01]). Given these outcomes, we retained our original model ordering.

**Table S1.** Substance use metrics by data collection wave.

|  | **Wave 1 (*n* = 153)** | **Wave 2 (*n* = 140)** |
| --- | --- | --- |
| **Total** |  |  |
| Lifetime (no use/use) | 48/105 (69% Use) | - |
| Use between W1 and W2 (no use/use) | - | 64/76 (54% Use) |
| Age of first use *M*±*SD* (range) | 12.9 ± 2.36 (4-16) | - |
| Days of use* *M*±*SD* (range) | 24.1 ± 83.5 (0-365) | 24.1 ± 88.1 (0-455) |
| **Cannabis Use** |  |  |
| Lifetime (no/yes) | 121/32 (21% Use) | - |
| Use between W1 and W2 (no/yes) | - | 118/22 (16% Use) |
| Age of first use *M*±*SD* (range) | 14.3 ± 0.97 (12-16) | - |
| Days of use**M*±*SD* (range) | 10.8 ± 50.4 (0-365) | 13.51 ± 56.8 (0-455) |
| **E-cigarette Use** |  |  |
| Lifetime (no/yes) | 95/58 (38% Use) | - |
| Use between W1 and W2 (no/yes) | - | 110/30 (21% Use) |
| Age of first use *M*±*SD* (range) | 14.2 ± 1.12 (10-16) | - |
| Days of use* *M*±*SD* (range) | 10.33 ± 40.8 (0-365) | 10.03 ± 38.9 (0-256) |
| **Alcohol Use** |  |  |
| Lifetime (no/yes) | 67/86 (56% Use) | - |
| Use between W1 and W2 (no/yes) | - | 81/59 (42% Use) |
| Age of first use *M*±*SD* (range) | 11.9 ± 2.87 (4-16) | - |
| Days of use* *M*±*SD* (range) | 3.01 ± 14.9 (0-156) | 3.21 ± 13.5 (0-128) |

**Note.** Baseline SU rates (Wave 1) were characterized between March 2018 through December 2019) and follow-up SU rates (Wave 2) between June 2019 to June 2021. * For W1: days of use 12 months prior to baseline assessment. For W2: days of use between W1 and W2 assessments.

We focused on cannabis, e-cigarettes, and alcohol, given these are the most commonly used substances among teens (16). Our data (lifetime use at baseline) align with national trends with the highest rates being reported for alcohol (56%), e-cigarette (38%), and cannabis use (21%) followed by other forms of nicotine delivery (i.e., hookah: 8%, cigarettes: 8%, and cigars 2%). Use of other substances was low (i.e., amphetamine type stimulants: 3%, sedatives/narcotics: 3%) or not reported in our cohort (e.g., psychedelics, cocaine, heroin, inhalants).

**Table S2.** Descriptives and correlations across study variables.

| **Variable** | M | SD | 1 | 2 | 3 | 4 | 5 | 6 | 7 | 8 | 9 | 10 | 11 | 12 | 13 | 14 | 15 | 16 | 17 |
| --- | --- | --- | --- | --- | --- | --- | --- | --- | --- | --- | --- | --- | --- | --- | --- | --- | --- | --- | --- |
| 1. Age (W1a) | 14.92 | 0.69 |  |  |  |  |  |  |  |  |  |  |  |  |  |  |  |  |  |
| 2. Biological Sex ^a^ (W1a) | - | - | .02 |  |  |  |  |  |  |  |  |  |  |  |  |  |  |  |  |
| 3. Race ^b^ (W1a) | - | - | -.04 | .07 |  |  |  |  |  |  |  |  |  |  |  |  |  |  |  |
| 4. Hispanic/Latinx ^c^ (W1a) | - | - | .13 | -.09 | -.16 |  |  |  |  |  |  |  |  |  |  |  |  |  |  |
| 5. Days of E-cigarette Use (W1a) | 10.33 | 40.81 | .09 | .11 | -.09 | -.08 |  |  |  |  |  |  |  |  |  |  |  |  |  |
| 6. Days of E-cigarette Use (W2) | 10.03 | 38.92 | .03 | .08 | -.07 | -.08 | **.20^*^** |  |  |  |  |  |  |  |  |  |  |  |  |
| 7. Days of Cannabis Use (W1a) | 10.77 | 50.43 | .15 | .15 | -.08 | .07 | **.37^**^** | **.17^*^** |  |  |  |  |  |  |  |  |  |  |  |
| 8. Days of Cannabis Use (W2) | 13.51 | 56.83 | .15 | .12 | -.07 | .07 | **.21^*^** | **.48^**^** | **.76^**^** |  |  |  |  |  |  |  |  |  |  |
| 9. Days of Alcohol Use (W1a) | 3.01 | 14.86 | **.17*** | .01 | -.07 | -.04 | **.24^**^** | .01 | **.49^**^** | **.58^**^** |  |  |  |  |  |  |  |  |  |
| 10. Days of Alcohol Use (W2) | 3.21 | 13.50 | .05 | .02 | -.08 | .09 | **.23^**^** | .08 | **.36**** | **.40^**^** | .09 |  |  |  |  |  |  |  |  |
| 11. Impulsivity ^1^ (W1b) | 41.23 | 8.15 | -.05 | .02 | .05 | .02 | **.24^**^** | .14 | **.21*** | .11 | .03 | .06 |  |  |  |  |  |  |  |
| 12. Externalizing ^2^ (W2) | 8.81 | 6.08 | -.04 | .04 | .09 | -.06 | .07 | **.34^**^** | **.17*** | **.28^**^** | .04 | .10 | **.35^**^** |  |  |  |  |  |  |
| 13. MID RT ^3^ (W1b, gains) | 0.30 | 0.04 | **-.19^*^** | **-.25^**^** | **-.19^*^** | **-.18^*^** | .09 | .15 | .04 | .01 | .03 | .08 | .10 | **.21^*^** |  |  |  |  |  |
| 14. MID HR ^4^ (W1b, gains) | 0.70 | 0.07 | .16 | .15 | .14 | .06 | -.07 | .00 | .03 | .05 | .03 | -.11 | .06 | -.00 | **-.39^**^** |  |  |  |  |
| 15. R. Striatum (W1b) | 0.07 | 0.12 | .02 | .07 | -.02 | .08 | .04 | .11 | .06 | .14 | .04 | -.01 | -.11 | -.03 | **-.29^**^** | .07 |  |  |  |
| 16. L. Striatum (W1b) | 0.09 | 0.19 | .08 | .02 | .01 | .11 | .02 | .15 | -.01 | .06 | -.03 | .01 | **-.18*** | -.01 | **-.35^**^** | .02 | **.72^**^** |  |  |
| 17. R. SFG (W1b) | 0.43 | 0.38 | .07 | .05 | .09 | .16* | -.12 | .02 | .08 | .04 | .01 | -.07 | -.13 | -.01 | **-.39^**^** | .15 | **.40^**^** | **.45^**^** |  |
| 18. L. Insula (W1b) | 0.09 | 0.15 | -.03 | **-.19^*^** | **-.22^**^** | .04 | -.15 | -.10 | -.06 | -.09 | -.02 | -.08 | -.06 | -.04 | .06 | .08 | -.01 | -.04 | .10 |

**Note.** M (mean) and SD (standard deviation) for the participant subsample completing both waves 1 and 2 (n=140). W1a = Wave 1, Visit 1. W1b = Wave 1, Visit 2. W2 = Wave 2. ^a^ Female = 0 (49%, n = 68), Male = 1 (51%, n = 72); ^b^ White = 0 (83%, n = 116), Non-White = (17%, n = 24); ^c^ Non-Hispanic/Latino = 0 (12%, n = 17), Hispanic/Latino(a) = 1 (88%, n = 123); ^1^ UPPS-P total score; ^2^ ASEBA YSR Externalizing subscale total score; ^3^ Target response times on gain trials; ^4^ Hit rates on gain trials; L. Striatum (caudate), R. Striatum (caudate), and SFG ROIs are from anticipatory gain cues; L. Insula ROI is from anticipatory neutral cues. ***** p < 0.05. ****** p < 0.01.

Covariate selection (for serial mediation models). We considered bivariate correlations among study variables to aid covariate selection. Specifically, age correlated with alcohol use at W1 and age, biological sex, race, and ethnicity correlated with behavioral reward-responsivity (i.e., gain-trial RT). Given these interrelations, our serial mediation models included the following covariates: age, sex, race, ethnicity, and substance-specific days of use at W1(see also: **Supplemental Tables S6-S8** for results covarying for multiple substance use).

Baseline (W1) and follow-up (W2) SU correlations. Noteworthy, W2 data collection took place during implementation of COVID-19 social distancing and remote learning practices. Nationally, these practices have been linked with reduced adolescent SU rates (17-20) which have persisted through the 2024 (16). These COVID-19 practices likely impacted psychosocial factors influencing SU (e.g., peer, parental interactions) and substance availability/access (20-22). In 2021, Monitoring the Future (MTF) Study data indicated an appreciable reduction in annual SU rates (cannabis: 17%, e-cigarette: 20%, alcohol: 29%) relative to the prior year’s report (cannabis: 28%, e-cigarette: 31%, alcohol: 41%). Our sample’s data generally align with these contemporaneous national trends where cannabis (16%) and e-cigarette use (21%) rates were lower at W2 (2019-2021) compared to W1 (2018-2019; cannabis: 21%, e-cigarette: 35%). When considering alcohol, our sample showed an increase in annual use rates from W1 (33%) to W2 (42%). These COVID-19 factors as well as changing regulatory policies (e.g., Tobacco21 in December 2019) likely impacted the correlations between W1 and W2 SU.

Nonetheless, we did observe many expected correlations when considering SU days at W1 and W2. Regarding within-substance interrelations, significant correlations between W1 and W2 were observed for cannabis (r[138]=0.76, p<0.001) and e-cigarette use (r[138]=0.2, p=0.017). While a similar W1-W2 correlation was not detected for alcohol use (r[138]=0.09, p=0.29), the direction of the relationship was in the expected direction. Regarding between-substance correlations, W1 cannabis use correlated with W2 e-cigarette (r[138]=0.17, p=0.04) and W2 alcohol use (r[138]=0.36, p<0.01). W1 e-cigarette use correlated with W2 cannabis (r[138]=0.21, p=0.013) and alcohol use (r[138]=0.23, p=0.006). W1 alcohol use correlated with W2 cannabis (r[138]=0.58 p<0.001), but not W2 e-cigarette use (r[138]=0.01, p=0.9).

**Table S3.** Serial mediation outcomes linking brain, impulsivity, externalizing, and cannabis use.

|  | Impulsivity W1b (M_1_) | | | | Externalizing W2 (M_2_) | | | | Cannabis Use W2 (Y) | | | | |
| --- | --- | --- | --- | --- | --- | --- | --- | --- | --- | --- | --- | --- | --- |
| **L. Striatum** | Coefficient | (95% CI) | *SE* | *p-v*alue | Coefficient | (95% CI) | *SE* | *p-v*alue | Coefficient | (95% CI) | *SE* | *p-v*alue | |
| Intercept | 0.99 | (-2.63, 4.61) | 1.83 | 0.59 | 1.03 | (-2.43, 4.50) | 1.75 | 0.56 | -0.38 | (-2.73, 1.97) | 1.19 | 0.75 | |
| Age | -0.08 | (-0.32, 0.17) | 0.12 | 0.10 | -0.06 | (-0.29, 0.17) | 0.12 | 0.61 | 0.02 | (-0.13, 0.18) | 0.08 | 0.77 | |
| Sex ^a^ | 0.04 | (-0.30, 0.37) | 0.17 | 0.83 | -0.004 | (-0.32, 0.32) | 0.16 | 0.98 | 0.04 | (-0.17, 0.26) | 0.11 | 0.69 | |
| Race ^b^ | 0.28 | (-0.23, 0.79) | 0.26 | 0.28 | 0.21 | (-0.28, 0.70) | 0.25 | 0.40 | -0.08 | (-0.04, 0.25) | 0.17 | 0.63 | |
| Hispanic/Latinx ^c^ | 0.06 | (-0.38, 0.51) | 0.23 | 0.78 | -0.16 | (-0.59, 0.27) | 0.22 | 0.47 | 0.06 | (-0.23, 0.35) | 0.15 | 0.67 | |
| Days of Cannabis Use (W1a) | 0.17 | (-0.03, 0.37) | 0.10 | 0.10 | 0.17 | (-0.03, 0.36) | 0.10 | 0.09 | **0.86** | **(0.72, 0.99)** | **0.07** | **<0.001** | |
| L. Striatum (W1b) | **-0.20** | **(-0.37, -0.03)** | **0.08** | **0.02** | 0.08 | (-0.08, 0.24) | 0.08 | 0.32 | 0.08 | (-0.03, 0.19) | 0.06 | 0.15 | |
| Impulsivity ^1^ (W1b) | - | - | - | - | **0.34** | **(0.17, 0.50)** | **0.08** | **<0.001** | -0.03 | (-0.15, 0.08) | 0.06 | 0.62 | |
| Externalizing ^2^ (W2) | - | - | - | - | - | - | - | - | **0.17** | **(0.06, 0.29)** | **0.06** | **0.004** | |
| Indirect Effect | 0.006 | (-0.02, 0.03) | 0.01 | - | 0.01 | (-0.02, 0.06) | 0.02 | - | **-0.01** | **(-0.03, -0.001)** | **0.01** | - | |
|  | ***R^2^* = 0.07** | | | | ***R^2^* = 0.16** | | | | ***R^2^* = 0.62** | | | | |
| **R. Striatum** | Impulsivity W1b (M_1_) | | | | Externalizing W2 (M_2_) | | | | Cannabis Use W2 (Y) | | | | |
| Intercept | 1.27 | (-2.39, 4.93) | 1.85 | 0.49 | 0.91 | (-2.56, 4.38) | 1.75 | 0.61 | -0.43 | (-2.75, 1.89) | 1.17 | 0.71 | |
| Age | -0.09 | (-0.34, 0.15) | 0.12 | 0.46 | -0.05 | (-0.29, 0.18) | 0.12 | 0.65 | 0.03 | (-0.13, 0.18) | 0.08 | 0.73 | |
| Sex ^a^ | 0.05 | (-0.29, 0.39) | 0.17 | 0.79 | -0.01 | (-0.33, 0.32) | 0.16 | 0.97 | 0.04 | (-0.18, 0.25) | 0.11 | 0.73 | |
| Race ^b^ | 0.27 | (-0.25, 0.79) | 0.26 | 0.30 | 0.21 | (-0.28, 0.70) | 0.25 | 0.40 | -0.07 | (-0.40, 0.26) | 0.17 | 0.68 | |
| Hispanic/Latinx ^c^ | 0.04 | (-0.42, 0.49) | 0.23 | 0.87 | -0.14 | (-0.57, 0.29) | 0.22 | 0.52 | 0.06 | (-0.23, 0.35) | 0.15 | 0.67 | |
| Days of Cannabis Use (W1a) | 0.18 | (-0.02, 0.39) | 0.10 | 0.07 | 0.16 | (-0.03, 0.36) | 0.10 | 0.10 | **0.85** | **(0.72, 0.98)** | **0.07** | **<0.001** | |
| R. Striatum (W1b) | -0.12 | (-0.29, 0.05) | 0.09 | 0.17 | 0.02 | (-0.15, 0.18) | 0.08 | 0.85 | 0.12 | (-0.003, 0.26) | 0.11 | 0.13 | |
| Impulsivity ^1^ (W1b) | - | - | - | - | **0.33** | **(0.16, 0.49)** | **0.08** | **<0.001** | -0.03 | (-0.15, 0.08) | 0.06 | 0.56 | |
| Externalizing ^2^ (W2) | - | - | - | - | - | - | - | - | **0.18** | **(0.06, 0.29)** | **0.06** | **0.003** | |
| Indirect Effect | 0.004 | (-0.01, 0.02) | 0.01 | - | 0.003 | (-0.03, 0.04) | 0.02 | - | -0.01 | (-0.02, 0.002) | 0.01 | - | |
|  | ***R^2^* = 0.05** | | | | ***R^2^* = 0.15** | | | | ***R^2^* = 0.62** | | | | |
| **Note.** W1a = Wave 1, Visit 1. W1b = Wave 1, Visit 2. W2 = Wave 2. ^a^ Female=0, Male=1. ^b^ White=0, Non-White=1. ^c^ non-Hispanic/Latino(a)=0, Hispanic/Latino(a)=1. ^1^ UPPS-P total score; ^2^ ASEBA YSR Externalizing composite score (with three SU items from the rule-breaking subscale removed). L. Striatum (caudate) and R. Striatum ***β’s*** are from anticipatory gain cues, see also main text **Figure 2**. Indirect Effect 1 = Brain ROI (X) → UPPS-P total score (M1) → Cannabis use days (Y). Indirect Effect 2 = Brain ROI (X) → YSR externalizing score (M2) → Cannabis use days (Y). Indirect Effect 3 = Brain ROI (X) → UPPS-P total score (M1) → YSR externalizing score (M2) → Cannabis use days (Y). Coefficients represent standardized effects. | | | | | | | | | | | | |  |

**Table S4.** Serial mediation outcomes linking brain, impulsivity, externalizing, and e-cigarette use.

|  | Impulsivity W1b (M_1_) | | | | Externalizing W2 (M_2_) | | | | E-cigarette Use W2 (Y) | | | |
| --- | --- | --- | --- | --- | --- | --- | --- | --- | --- | --- | --- | --- |
| **L. Striatum** | Coefficient | (95% CI) | *SE* | *p-v*alue | Coefficient | (95% CI) | *SE* | *p-v*alue | Coefficient | (95% CI) | *SE* | *p-v*alue |
| Intercept | 0.90 | (-2.61, 4.41) | 1.78 | 0.61 | 0.45 | (-3.01, 3.91) | 1.75 | 0.80 | -0.35 | (-3.72, 3.03) | 1.71 | 0.84 |
| Age | -0.07 | (-0.31, 0.16) | 0.12 | 0.54 | -0.02 | (-0.26, 0.21) | 0.12 | 0.84 | 0.03 | (-0.19, 0.26) | 0.12 | 0.77 |
| Sex ^a^ | 0.04 | (-0.29, 0.36) | 0.17 | 0.83 | 0.03 | (-0.29, 0.36) | 0.16 | 0.84 | 0.10 | (-0.21, 0.42) | 0.16 | 0.52 |
| Race ^b^ | 0.32 | (-0.18, 0.83) | 0.25 | 0.21 | 0.17 | (-0.32, 0.67) | 0.25 | 0.49 | -0.30 | (-0.79, 0.18) | 0.25 | 0.22 |
| Hispanic/Latinx ^c^ | 0.16 | (-0.29, 0.60) | 0.22 | 0.49 | -0.14 | (-0.58, 0.30) | 0.22 | 0.53 | -0.20 | (-0.63, 0.23) | 0.22 | 0.35 |
| Days of E-Cig. Use (W1a) | **0.23** | **(0.06, 0.40)** | **0.08** | **0.007** | -0.002 | (-0.17, 0.17) | 0.09 | 0.99 | 0.14 | (-0.02, 0.31) | 0.08 | 0.09 |
| L. Striatum (W1b) | **-0.21** | **(-0.37, -0.05)** | **0.08** | **0.01** | 0.08 | (-0.09, 0.24) | 0.08 | 0.35 | **0.17** | **(0.005, 0.33)** | **0.08** | **0.04** |
| Impulsivity ^1^ (W1b) | - | - | - | - | **0.36** | **(0.19, 0.53)** | **0.09** | **<0.001** | 0.05 | (-0.13, 0.22) | 0.09 | 0.60 |
| Externalizing ^2^ (W2) | - | - | - | - | - | - | - | - | **0.31** | **(0.15, 0.48)** | **0.09** | **<0.001** |
| Indirect Effect | -0.01 | (-0.04, 0.01) | 0.01 | - | 0.02 | (-0.04, 0.10) | 0.03 | - | **-0.02** | **(-0.06, -0.003)** | **0.01** | - |
|  | ***R* = 0.10** | | | | ***R* = 0.14** | | | | ***R* = 0.18** | | | |
| **R. Striatum** | Impulsivity W1b (M_1_) | | | | Externalizing W2 (M_2_) | | | | E-cigarette Use W2 (Y) | | | |
| Intercept | 1.14 | (-2.42, 4.70) | 1.80 | 0.53 | 0.36 | (-3.10, 3.82) | 1.75 | 0.84 | -0.48 | (-3.88, 2.91) | 1.72 | 0.78 |
| Age | -0.09 | (-0.33, 0.15) | 0.12 | 0.46 | -0.02 | (-0.25, 0.21) | 0.12 | 0.87 | 0.04 | (-0.19, 0.27) | 0.12 | 0.72 |
| Sex ^a^ | 0.05 | (-0.28, 0.38) | 0.17 | 0.77 | 0.03 | (-0.29, 0.35) | 0.16 | 0.85 | 0.09 | (-0.23, 0.41) | 0.16 | 0.57 |
| Race ^b^ | 0.31 | (-0.20, 0.82) | 0.26 | 0.23 | 0.18 | (-0.32, 0.68) | 0.25 | 0.48 | -0.29 | (-0.78, 0.20) | 0.25 | 0.25 |
| Hispanic/Latinx ^c^ | 0.13 | (-0.32, 0.58) | 0.23 | 0.58 | -0.12 | (-0.58, 0.31) | 0.22 | 0.58 | -0.18 | (-0.61, 0.25) | 0.22 | 0.41 |
| Days of E-Cig. Use (W1a) | **0.23** | **(0.06, 0.40)** | **0.09** | **0.009** | **0.003** | **(-0.17, 0.17)** | **0.09** | **0.97** | 0.15 | (-0.02, 0.31) | 0.08 | 0.08 |
| R. Striatum (W1b) | -0.12 | (-0.29, 0.05) | 0.09 | 0.16 | 0.02 | (-0.15, 0.19) | 0.08 | 0.82 | 0.13 | (-0.04, 0.29) | 0.08 | 0.13 |
| Impulsivity ^1^ (W1b) | - | - | - | - | **0.35** | **(0.18, 0.51)** | **0.08** | **<0.001** | 0.02 | (-0.15, 0.19) | 0.09 | 0.82 |
| Externalizing ^2^ (W2) | - | - | - | - | - | - | - | - | **0.33** | **(0.16, 0.49)** | **0.09** | **<0.001** |
| Indirect Effect | -0.003 | (-0.02, 0.01) | 0.01 | - | 0.006 | (-0.05, 0.08) | 0.03 | - | -0.01 | (-0.05, 0.004) | 0.01 | - |
|  | ***R^2^* = 0.07** | | | | ***R^2^* = 0.13** | | | | ***R^2^* = 0.17** | | | |
| **Note.** W1a = Wave 1, Visit 1. W1b = Wave 1, Visit 2. W2 = Wave 2. ^a^ Female=0, Male=1. ^b^ White=0, Non-White=1. ^c^ non-Hispanic/Latino(a)=0, Hispanic/Latino(a)=1. ^1^ UPPS-P total score; ^2^ ASEBA YSR Externalizing composite score (with three SU items from the rule-breaking subscale removed). L. Striatum (caudate) and R. Striatum ***β’s*** are from anticipatory gain cues, see also main text **Figure 2**. Indirect Effect 1 = Brain ROI (X) → UPPS-P total score (M1) → E-cigarette use days (Y). Indirect Effect 2 = Brain ROI (X) → YSR externalizing score (M2) → E-cigarette use days (Y). Indirect Effect 3 = Brain ROI (X) → UPPS-P total score (M1) → YSR externalizing score (M2) → E-cigarette use days (Y). Coefficients represent standardized effects.  Partial mediation interpretation. In the e-cigarette model, opposing signs across paths were present such that striatal activity was negatively associated with impulsivity (which in turn was positively associated with externalizing), yielding a “negative indirect effect”, while striatal activity showed a “positive direct effect” with subsequent e-cigarette use. We interpret this pattern as consistent with multiple, partially independent pathways linking reward-related striatal activity with adolescent nicotine use.  The *negative* ***indirect pathway*** is consistent with a reward-deficit/motivational-inefficiency account where less striatal activity relates to greater disinhibitory tendencies (impulsivity → externalizing), which may increase risk for trying or escalating nicotine use as adolescents seek higher-intensity stimulation or immediate reinforcement (e.g., (23, 24)).  The ***positive direct effect*** suggests an alternative pathway and associated constructs not fully captured in our models. Speculatively, some possible unmodeled constructs linking more striatal activity with e-cigarette use may be addiction severity, susceptibility to social influences, or aspects of learning/conditioning (e.g., drug-cue reactivity). For example, one possibility is that striatal activity may link with nicotine dependence severity driving more compulsive use independent of the modeled impulsivity and externalizing constructs (25-27). Another possibility is that striatal activity may link with heightened sensitivity to social rewards leading to greater susceptibility to peer influences driving use (28-30). A third possibility is that striatal activity may relate to the formation of learned/conditioned stimulus-reward associations which in the context of e-cigarette use may involve cue-reactivity (e.g., devices, flavors, scents, contexts) promoting use beyond the disinhibitory pathway delineated. | | | | | | | | | | | | |

**Table S5.** Serial mediation outcomes linking brain, impulsivity, externalizing, and alcohol use.

|  | Impulsivity W1b (M_1_) | | | | Externalizing W2 (M_2_) | | | | Alcohol Use W2 (Y) | | | | |
| --- | --- | --- | --- | --- | --- | --- | --- | --- | --- | --- | --- | --- | --- |
| **L. Striatum** | Coefficient | (95% CI) | *SE* | *p-v*alue | Coefficient | (95% CI) | *SE* | *p-v*alue | Coefficient | (95% CI) | *SE* | *p-v*alue |  |
| Intercept | 0.57 | (-3.07, 4.22) | 1.84 | 0.76 | 0.60 | (-2.89, 4.09) | 1.76 | 0.74 | -0.78 | (-4.48, 2.93) | 1.88 | 0.68 |  |
| Age | -0.05 | (-0.30, 0.20) | 0.12 | 0.69 | -0.03 | (-0.27, 0.20) | 0.12 | 0.77 | 0.04 | (-0.21, 0.29) | 0.13 | 0.76 |  |
| Sex ^a^ | 0.08 | (-0.26, 0.41) | 0.17 | 0.66 | 0.03 | (-0.29, 0.35) | 0.16 | 0.84 | 0.06 | (-0.28, 0.40) | 0.17 | 0.74 |  |
| Race ^b^ | 0.26 | (-0.26, 0.78) | 0.26 | 0.32 | 0.18 | (-0.31, 0.68) | 0.25 | 0.47 | -0.22 | (-0.75, 0.31) | 0.27 | 0.41 |  |
| Hispanic/Latinx ^c^ | 0.09 | (-0.36, 0.55) | 0.23 | 0.69 | -0.13 | (-0.57, 0.30) | 0.22 | 0.55 | 0.25 | (-0.22, 0.71) | 0.23 | 0.29 |  |
| Days of Alcohol Use (W1a) | 0.04 | (-0.12, 0.20) | 0.08 | 0.62 | 0.04 | (-0.12, 0.19) | 0.08 | 0.65 | 0.07 | (-0.09, 0.24) | 0.08 | 0.39 |  |
| L. Striatum (W1b) | **-0.21** | **(-0.37, -0.04)** | **0.08** | **0.02** | 0.08 | (-0.09, 0.24) | 0.08 | 0.34 | 0.004 | (-0.17, 0.18) | 0.09 | 0.97 |  |
| Impulsivity ^1^ (W1b) | - | - | - | - | **0.36** | **(0.19, 0.52)** | **0.08** | **<0.001** | 0.03 | (-0.16, 0.21) | 0.09 | 0.78 |  |
| Externalizing ^2^ (W2) | - | - | - | - | - | - | - | - | 0.10 | (-0.08, 0.28) | 0.09 | 0.29 |  |
| Indirect Effect | -0.01 | (-0.04, 0.03) | 0.02 | - | 0.01 | (-0.02, 0.05) | 0.02 | - | -0.01 | (-0.03, 0.003) | 0.01 | - |  |
|  | ***R* = 0.06** | | | | ***R* = 0.14** | | | | ***R*= 0.03** | | | | |
| **R. Striatum** | Impulsivity W1b (M_1_) | | | | Externalizing W2 (M_2_) | | | | Alcohol Use W2 (Y) | | | | |
| Intercept | 0.86 | (-2.83, 4.55) | 1.87 | 0.64 | 0.48 | (-3.01, 3.97) | 1.77 | 0.79 | -0.80 | (-4.49, 2.90) | 1.87 | 0.67 |  |
| Age | -0.07 | (-0.32, 0.18) | 0.13 | 0.59 | -0.03 | (-0.26, 0.21) | 0.12 | 0.82 | 0.04 | (-0.21, 0.29) | 0.13 | 0.76 |  |
| Sex ^a^ | 0.09 | (-0.25, 0.43) | 0.17 | 0.61 | 0.03 | (-0.29, 0.35) | 0.16 | 0.85 | 0.06 | (-0.28, 0.40) | 0.17 | 0.74 |  |
| Race ^b^ | 0.25 | (-0.27, 0.77) | 0.26 | 0.34 | 0.19 | (-0.31, 0.68) | 0.25 | 0.46 | -0.22 | (-0.75, 0.30) | 0.27 | 0.41 |  |
| Hispanic/Latinx ^c^ | 0.07 | (-0.39, 0.53) | 0.23 | 0.77 | -0.12 | (-0.55, 0.32) | 0.22 | 0.59 | 0.25 | (-0.21, 0.71) | 0.23 | 0.28 |  |
| Days of Alcohol Use (W1a) | 0.05 | (-0.11, 0.22) | 0.08 | 0.53 | 0.03 | (-0.12, 0.19) | 0.08 | 0.67 | 0.07 | (-0.09, 0.24) | 0.08 | 0.38 |  |
| R. Striatum (W1b) | -0.12 | (-0.30, 0.05) | 0.09 | 0.17 | 0.02 | (-0.15, 0.18) | 0.08 | 0.84 | -0.03 | (-0.20, 0.15) | 0.09 | 0.78 |  |
| Impulsivity ^1^ (W1b) | - | - | - | - | **0.34** | **(0.18, 0.51)** | **0.08** | **<0.001** | 0.02 | (-0.16, 0.21) | 0.09 | 0.80 |  |
| Externalizing ^2^ (W2) | - | - | - | - | - | - | - | - | 0.10 | (-0.08, 0.28) | 0.09 | 0.28 |  |
| Indirect Effect | -0.003 | (-0.03, 0.02) | 0.01 | - | 0.002 | (-0.03, 0.03) | 0.01 | - | -0.004 | (-0.02, 0.002) | 0.01 | - |  |
|  | ***R^2^* = 0.03** | | | | ***R^2^* = 0.13** | | | | ***R^2^* = 0.04** | | | | |

**Note.** W1a = Wave 1, Visit 1. W1b = Wave 1, Visit 2. W2 = Wave 2. ^a^ Female=0, Male=1. ^b^ White=0, Non-White=1. ^c^ non-Hispanic/Latino(a)=0, Hispanic/Latino(a)=1. ^1^ UPPS-P total score; ^2^ ASEBA YSR Externalizing composite score (with three SU items from the rule-breaking subscale removed). L. Striatum (caudate) and R. Striatum ***β’s*** are from anticipatory gain cues, see also main text **Figure 2**. Indirect Effect 1 = Brain ROI (X) → UPPS-P total score (M1) → Alcohol use days (Y). Indirect Effect 2 = Brain ROI (X) → YSR externalizing score (M2) → Alcohol use days (Y). Indirect Effect 3 = Brain ROI (X) → UPPS-P total score (M1) → YSR externalizing score (M2) → Alcohol use days (Y). Coefficients represent standardized effects.

**Table S6.** Serial mediation outcomes linking brain, impulsivity, externalizing, and cannabis use controlling for multiple substances.

|  | Impulsivity W1b (M_1_) | | | | Externalizing W2 (M_2_) | | | | Cannabis Use W2 (Y) | | | |
| --- | --- | --- | --- | --- | --- | --- | --- | --- | --- | --- | --- | --- |
| **L. Striatum** | Coefficient | (95% CI) | *SE* | *p-v*alue | Coefficient | (95% CI) | *SE* | *p-v*alue | Coefficient | (95% CI) | *SE* | *p-v*alue |
| Intercept | 1.12 | (-2.46, 4.69) | 1.81 | 0.54 | 0.91 | (-2.59, 4.41) | 1.77 | 0.61 | -0.11 | (-2.40, 2.18) | 1.16 | 0.92 |
| Age | -0.09 | (-0.33, 0.16) | 0.12 | 0.49 | -0.05 | (-0.29, 0.18) | 0.12 | 0.67 | 0.001 | (-0.15, 0.16) | 0.08 | 0.99 |
| Sex ^a^ | -0.002 | (-0.33, 0.33) | 0.17 | 0.99 | -0.01 | (-0.33, 0.31) | 0.16 | 0.95 | 0.07 | (-0.14, 0.28) | 0.11 | 0.50 |
| Race ^b^ | 0.33 | (-0.18, 0.83) | 0.25 | 0.20 | 0.19 | (-0.30, 0.69) | 0.25 | 0.44 | -0.06 | (-0.39, 0.26) | 0.16 | 0.69 |
| Hispanic/Latinx ^c^ | 0.11 | (-0.33, 0.56) | 0.23 | 0.62 | -0.18 | (-0.62, 0.25) | 0.22 | 0.41 | 0.12 | (-0.17, 0.40) | 0.15 | 0.42 |
| Days of Cannabis Use (W1a) | 0.18 | (-0.07, 0.42) | 0.12 | 0.15 | 0.22 | (-0.02, 0.46) | 0.12 | 0.08 | **0.70** | **(0.54, 0.86)** | **0.08** | **<0.001** |
| Days of E-Cig. Use (W1a) | **0.22** | **(0.05, 0.39)** | **0.09** | **0.01** | -0.02 | (-0.20, 0.15) | 0.09 | 0.78 | 0.002 | (-0.11, 0.12) | 0.06 | 0.97 |
| Days of Alcohol Use (W1a) | -0.9 | (-0.29, 0.11) | 0.10 | 0.36 | -0.06 | (-0.26, 0.13) | 0.10 | 0.52 | **0.20** | **(0.07, 0.33)** | **0.06** | **0.002** |
| L. Striatum (W1b) | **-0.20** | **(-0.37, -0.03)** | **0.08** | **0.02** | 0.08 | (-0.08, 0.25) | 0.08 | 0.32 | 0.08 | (-0.03, 0.19) | 0.05 | 0.14 |
| Impulsivity ^1^ (W1b) | - | - | - | - | **0.34** | **(0.17, 0.51)** | **0.09** | **<0.001** | -0.02 | (-0.14, 0.09) | 0.06 | 0.67 |
| Externalizing ^2^ (W2) | - | - | - | - | - | - | - | - | **0.18** | **(0.07, 0.30)** | **0.06** | **0.002** |
| Indirect Effect | 0.005 | (-0.02, 0.03) | 0.01 | - | 0.02 | (-0.02, 0.06) | 0.02 | - | **-0.01** | **(-0.03, -0.001)** | **0.01** | - |
|  | ***R^2^* = 0.12** | | | | ***R^2^* = 0.16** | | | | ***R^2^* = 0.64** | | | |

**Note.** W1a = Wave 1, Visit 1. W1b = Wave 1, Visit 2. W2 = Wave 2. ^a^ Female=0, Male=1. ^b^ White=0, Non-White=1. ^c^ non-Hispanic/Latino(a)=0, Hispanic/Latino(a)=1. ^1^ UPPS-P total score; ^2^ ASEBA YSR Externalizing composite score (with three SU items from the rule-breaking subscale removed). L. Striatum (caudate) and R. Striatum ***β’s*** are from anticipatory gain cues, see also main text **Figure 2**. Indirect Effect 1 = Brain ROI (X) → UPPS-P total score (M1) → Cannabis use days (Y). Indirect Effect 2 = Brain ROI (X) → YSR externalizing score (M2) → Cannabis use days (Y). Indirect Effect 3 = Brain ROI (X) → UPPS-P total score (M1) → YSR externalizing score (M2) → Cannabis use days (Y). Coefficients represent standardized effects.

For the models reported in the main text (**Figure 3**), we covaried for only substance-specific baseline use (e.g., W1 cannabis use in the cannabis model). As participants could have been using more than one substance, we conducted ancillary analyses estimating model parameters while controlling for other baseline SU (e.g., W1 cannabis, e-cigarette, and alcohol use in the cannabis model. Our primary outcomes and interpretations remained unchanged when including these additional SU covariates (c.f., Supplemental Table S6 and Table S3). Given these outcomes, we do not believe that polysubstance use complicated our interpretations, although we acknowledge that disentangling the unique neurobehavior contributions of specific substances remains a general challenge for adolescent SU studies (31, 32).

**Table S7.** Serial mediation outcomes linking brain, impulsivity, externalizing, and e-cigarette use controlling for multiple substances.

|  | Impulsivity W1b (M_1_) | | | | Externalizing W2 (M_2_) | | | | E-cigarette Use W2 (Y) | | | |
| --- | --- | --- | --- | --- | --- | --- | --- | --- | --- | --- | --- | --- |
| **L. Striatum** | Coefficient | (95% CI) | *SE* | *p-v*alue | Coefficient | (95% CI) | *SE* | *p-v*alue | Coefficient | (95% CI) | *SE* | *p-v*alue |
| Intercept | 1.12 | (-2.46, 4.69) | 1.81 | 0.54 | 0.91 | (-2.59, 4.41) | 1.77 | 0.61 | -0.23 | (-3.65, 3.20) | 1.73 | 0.90 |
| Age | -0.09 | (-0.33, 0.16) | 0.12 | 0.49 | -0.05 | (-0.29, 0.18) | 0.12 | 0.67 | 0.03 | (-0.20, 0.26) | 0.12 | 0.79 |
| Sex ^a^ | -0.002 | (-0.33, 0.33) | 0.17 | 0.99 | -0.01 | (-0.33, 0.31) | 0.16 | 0.95 | 0.06 | (-0.26, 0.38) | 0.16 | 0.70 |
| Race ^b^ | 0.33 | (-0.18, 0.83) | 0.25 | 0.20 | 0.19 | (-0.30, 0.69) | 0.25 | 0.44 | -0.30 | (-0.78, 0.19) | 0.25 | 0.23 |
| Hispanic/Latinx ^c^ | 0.11 | (-0.33, 0.56) | 0.23 | 0.62 | -0.18 | (-0.62, 0.25) | 0.22 | 0.41 | -0.25 | (-0.68, 0.18) | 0.22 | 0.25 |
| Days of E-Cig. Use (W1a) | **0.22** | **(0.05, 0.39)** | **0.09** | **0.01** | -0.02 | (-0.20, 0.15) | 0.09 | 0.78 | 0.14 | (-0.03, 0.31) | 0.09 | 0.10 |
| Days of Cannabis Use (W1a) | 0.18 | (-0.07, 0.42) | 0.12 | 0.15 | 0.22 | (-0.02, 0.46) | 0.12 | 0.08 | 0.20 | (-0.04, 0.44) | 0.12 | 0.10 |
| Days of Alcohol Use (W1a) | -0.9 | (-0.29, 0.11) | 0.10 | 0.36 | -0.06 | (-0.26, 0.13) | 0.10 | 0.52 | -0.15 | (-0.34, 0.04) | 0.10 | 0.12 |
| L. Striatum (W1b) | **-0.20** | **(-0.37, -0.03)** | **0.08** | **0.02** | 0.08 | (-0.08, 0.25) | 0.08 | 0.32 | **0.17** | **(0.01, 0.33)** | **0.08** | **0.04** |
| Impulsivity ^1^ (W1b) | - | - | - | - | **0.34** | **(0.17, 0.51)** | **0.09** | **<0.001** | 0.04 | (-0.14, 0.21) | 0.09 | 0.69 |
| Externalizing ^2^ (W2) | - | - | - | - | - | - | - | - | **0.29** | **(0.12, 0.46)** | **0.09** | **<0.001** |
| Indirect Effect | -0.01 | (-0.04, 0.02) | 0.01 | - | 0.02 | (-0.03, 0.10) | 0.03 | - | **-0.02** | **(-0.05, -0.002)** | **0.01** | **-** |
|  | ***R* = 0.12** | | | | ***R* = 0.16** | | | | ***R* = 0.20** | | | |

**Note.** W1a = Wave 1, Visit 1. W1b = Wave 1, Visit 2. W2 = Wave 2. ^a^ Female=0, Male=1. ^b^ White=0, Non-White=1. ^c^ non-Hispanic/Latino(a)=0, Hispanic/Latino(a)=1. ^1^ UPPS-P total score; ^2^ ASEBA YSR Externalizing composite score (with three SU items from the rule-breaking subscale removed). L. Striatum (caudate) and R. Striatum **β’s** are from anticipatory gain cues, see also main text **Figure 2**. Indirect Effect 1 = Brain ROI (X) → UPPS-P total score (M1) → E-cigarette use days (Y). Indirect Effect 2 = Brain ROI (X) → YSR externalizing score (M2) → E-cigarette use days (Y). Indirect Effect 3 = Brain ROI (X) → UPPS-P total score (M1) → YSR externalizing score (M2) → E-cigarette use days (Y). Coefficients represent standardized effects.

For the models reported in the main text (**Figure 3**), we covaried for only substance-specific baseline use (e.g., W1 e-cigarette use in the e-cigarette model). As participants could have been using more than one substance, we conducted ancillary analyses estimating model parameters while controlling for other baseline SU (e.g., W1 cannabis, e-cigarette, and alcohol use in the e-cigarette model). Our primary outcomes and interpretations remained unchanged when including these additional SU covariates (c.f., Supplemental Table S7 and Table S4). Given these outcomes, we do not believe that polysubstance use complicated our interpretations, although we acknowledge that disentangling the unique neurobehavior contributions of specific substances remains a general challenge for adolescent SU studies (31, 32).

**Table S8.** Serial mediation outcomes linking brain, impulsivity, externalizing, and alcohol use controlling for multiple substances.

|  | Impulsivity W1b (M_1_) | | | | Externalizing W2 (M_2_) | | | | | Alcohol Use W2 (Y) | | | | | |
| --- | --- | --- | --- | --- | --- | --- | --- | --- | --- | --- | --- | --- | --- | --- | --- |
| **L. Striatum** | Coefficient | (95% CI) | *SE* | *p-v*alue | Coefficient | (95% CI) | *SE* | *p-v*alue | Coefficient | | (95% CI) | *SE* | *p-v*alue | |  |
| Intercept | 1.12 | (-2.46, 4.69) | 1.81 | 0.54 | 0.91 | (-2.59, 4.41) | 1.77 | 0.61 | 0.37 | | (-3.06, 3.81) | 1.74 | 0.83 | |  |
| Age | -0.09 | (-0.33, 0.16) | 0.12 | 0.49 | -0.05 | (-0.29, 0.18) | 0.12 | 0.67 | -0.03 | | (-0.26, 0.20) | 0.12 | 0.80 | |  |
| Sex ^a^ | -0.002 | (-0.33, 0.33) | 0.17 | 0.99 | -0.01 | (-0.33, 0.31) | 0.16 | 0.95 | -0.09 | | (-0.40, 0.23) | 0.16 | 0.60 | |  |
| Race ^b^ | 0.33 | (-0.18, 0.83) | 0.25 | 0.20 | 0.19 | (-0.30, 0.69) | 0.25 | 0.44 | -0.10 | | (-0.59, 0.39) | 0.25 | 0.68 | |  |
| Hispanic/Latinx ^c^ | 0.11 | (-0.33, 0.56) | 0.23 | 0.62 | -0.18 | (-0.62, 0.25) | 0.22 | 0.41 | 0.19 | | (-0.24, 0.62) | 0.22 | 0.39 | |  |
| Days of Alcohol Use (W1a) | -0.9 | (-0.29, 0.11) | 0.10 | 0.36 | -0.06 | (-0.26, 0.13) | 0.10 | 0.52 | **-0.22** | | **(-0.41, 0.03)** | **0.10** | **0.03** | |  |
| Days of Cannabis Use (W1a) | 0.18 | (-0.07, 0.42) | 0.12 | 0.15 | 0.22 | (-0.02, 0.46) | 0.12 | 0.08 | **0.53** | | **(0.29, 0.77)** | **0.12** | **<0.001** | |  |
| Days of E-Cig. Use (W1a) | **0.22** | **(0.05, 0.39)** | **0.09** | **0.01** | -0.02 | (-0.20, 0.15) | 0.09 | 0.78 | **0.19** | | **(0.02, 0.36)** | **0.09** | **0.03** | |  |
| L. Striatum (W1b) | **-0.20** | **(-0.37, -0.03)** | **0.08** | **0.02** | 0.08 | (-0.08, 0.25) | 0.08 | 0.32 | 0.002 | | (-0.16, 0.16) | 0.08 | 0.98 | |  |
| Impulsivity ^1^ (W1b) | - | - | - | - | **0.34** | **(0.17, 0.51)** | **0.09** | **<0.001** | -0.05 | | (-0.22, 0.13) | 0.09 | 0.58 | |  |
| Externalizing ^2^ (W2) | - | - | - | - | - | - | - | - | 0.04 | | (-0.13, 0.21) | 0.09 | 0.62 | |  |
| Indirect Effect | 0.01 | (-0.03, 0.05) | 0.02 | - | 0.004 | (-0.01, 0.03) | 0.01 | - | -0.003 | | (-0.02, 0.01) | 0.01 | - | |  |
|  | ***R* = 0.12** | | | | ***R* = 0.16** | | | | ***R*= 0.20** | | | | |  |  |

**Note.** W1a = Wave 1, Visit 1. W1b = Wave 1, Visit 2. W2 = Wave 2. ^a^ Female=0, Male=1. ^b^ White=0, Non-White=1. ^c^ non-Hispanic/Latino(a)=0, Hispanic/Latino(a)=1. ^1^ UPPS-P total score; ^2^ ASEBA YSR Externalizing composite score (with three SU items from the rule-breaking subscale removed). L. Striatum (caudate) and R. Striatum ***β’s*** are from anticipatory gain cues, see also main text **Figure 2**. Indirect Effect 1 = Brain ROI (X) → UPPS-P total score (M1) → Alcohol use days (Y). Indirect Effect 2 = Brain ROI (X) → YSR externalizing score (M2) → Alcohol use days (Y). Indirect Effect 3 = Brain ROI (X) → UPPS-P total score (M1) → YSR externalizing score (M2) → Alcohol use days (Y). Coefficients represent standardized effects.

For the models reported in the main text (**Figure 3**), we covaried for only substance-specific baseline use (e.g., W1 alcohol use in the alchol model). As participants could have been using more than one substance, we conducted ancillary analyses estimating model parameters while controlling for other baseline SU (e.g., W1 cannabis, e-cigarette, and alcohol use in the alcohol model). Our primary outcomes and interpretations remained unchanged when including these additional SU covariates (c.f., Supplemental Table S8 and Table S5). Given these outcomes, we do not believe that polysubstance use complicated our interpretations, although we acknowledge that disentangling the unique neurobehavior contributions of specific substances remains a general challenge for adolescent SU studies (31, 32).


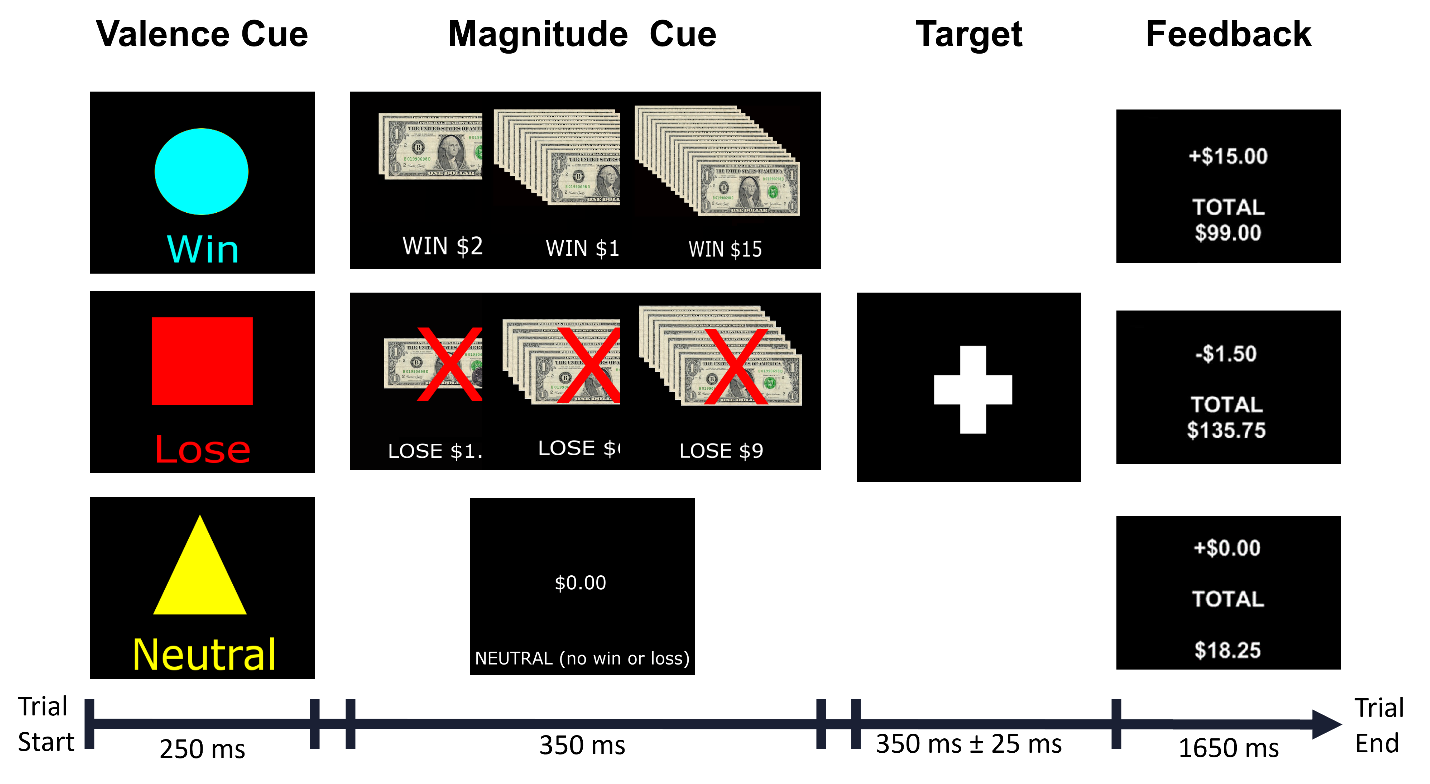


**Figure S1.** **MID task schematic**. Participants’ overall goal was to maximize gains and minimize losses by responding as quickly as possible when a visual target (white cross) appeared. Each MID trial consisted of four stimuli: a valence cue (350ms; blue circle = gain, red square = loss, yellow triangle = neutral), a magnitude cue (400ms; small, medium, large), a speeded target (variable duration), and performance feedback (1,500ms). Two variable interstimulus intervals (ISI, 800-3,200ms of fixation) separated the valence and magnitude cues, and the magnitude cues and target stimuli such that their durations summed to 4,000ms. A variable intertrial interval (ITI, 1,600-4,800ms of fixation) separated the current trial’s feedback display from the next trial’s initial valence cue. To introduce additional temporal jitter, null trials (3,200-4,800ms of fixation, n=64) were interleaved throughout the task. Targets were presented with a variable duration initialized at 350ms and adjusted dynamically in 25ms steps. The target response window was narrowed after hits and widened after misses to maintain ~66% accuracy. Feedback displayed the single-trial outcome (top of screen) and a running total of money accumulated over the task (bottom). The task included 168 trials across four, 8-min runs composed of 72 gain (42.9%), 72 loss (42.9%), and 24 neutral trials (14.3%). Gain and loss trials were each subdivided into 24 small, 24 medium, and 24 large magnitude trials.


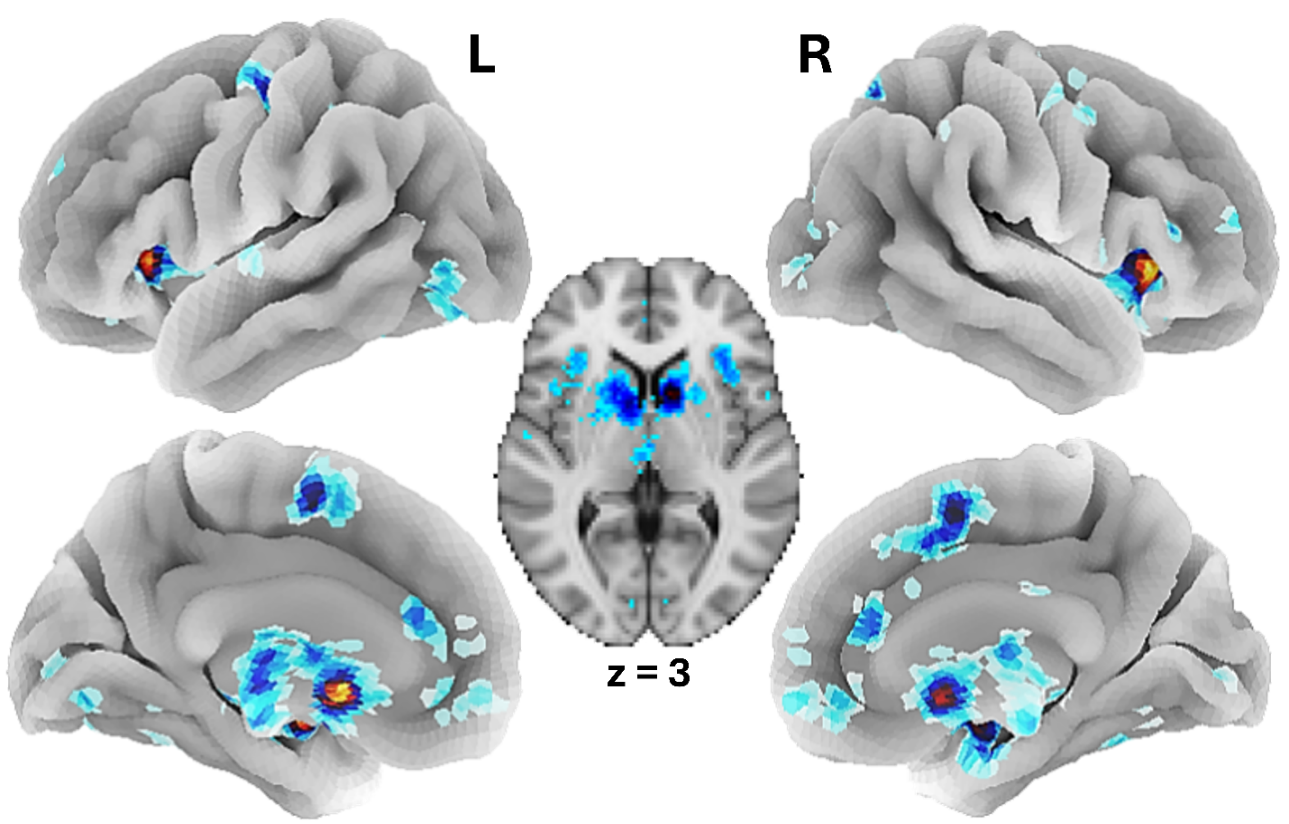


**Figure S2. Small volume corrected (SVC) analysis mask**. To **increase sensitivity while controlling for family-wise error,** we conducted small-volume corrected (SVC) analyses as opposed to whole-brain analyses. Specifically, we considered only voxels within a meta-analytically defined mask generated via Neurosynth (33) for the term “reward processing”. The meta-analysis contained 92 studies and was corrected for multiple comparisons using a FDR of 0.01 (33).

*
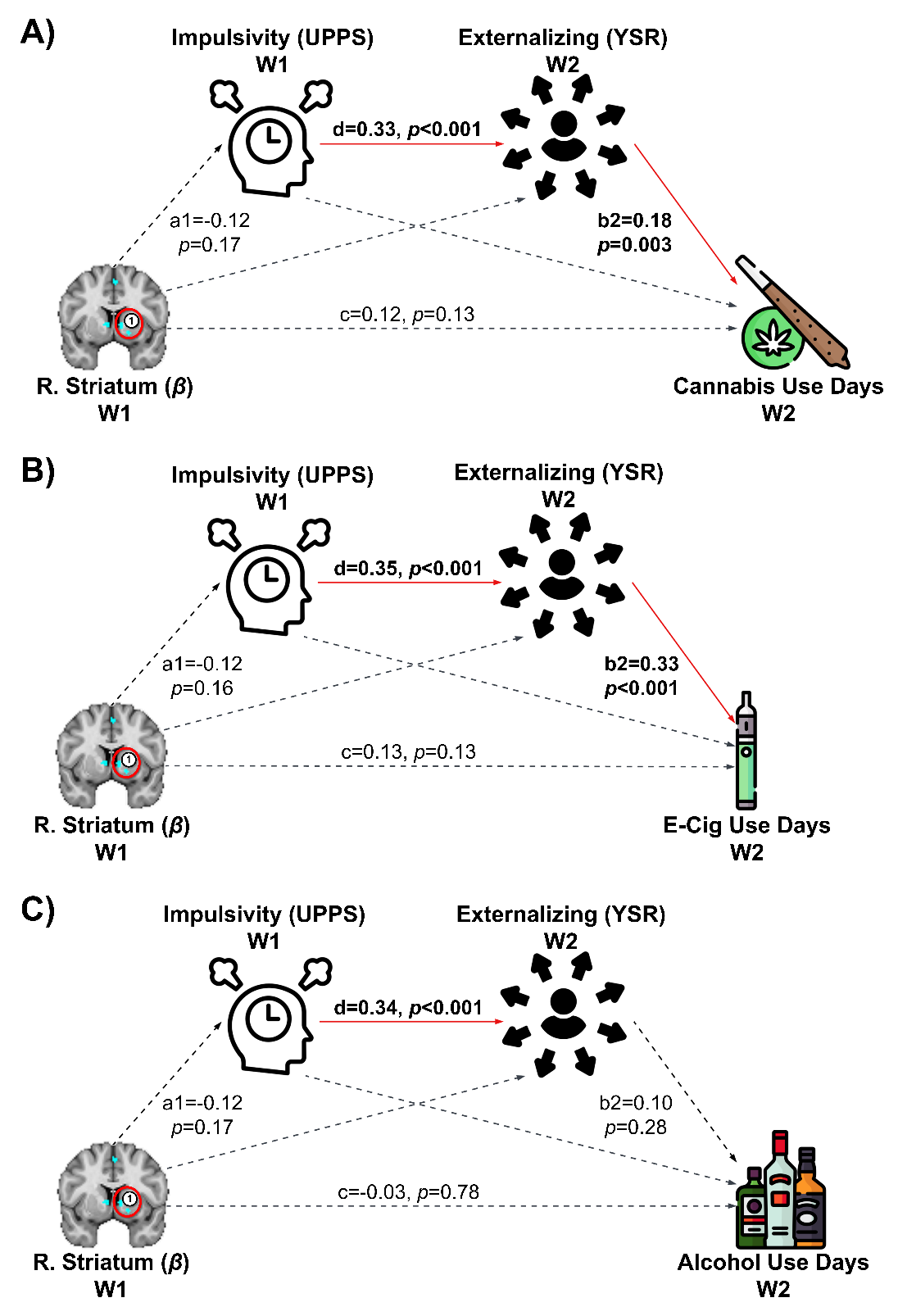
*

**Figure S3. Serial mediation models linking right striatal (caudate) activity, impulsivity, externalizing, and future substance use. A)** No direct or indirect effects linked right striatal activation during reward anticipation (Indirect Effect 3: -0.01, 95%CI[-0.02, 0.002]) with cannabis use (see Table S3, bottom). **B)** No direct or indirect effects linked right striatal activation during reward anticipation (Indirect Effect 3: -0.01, 95%CI [-0.05, 0.004]) with e-cigarette use (see Table S4, bottom). **C)** No direct or indirect effects linked right striatal activation during reward anticipation (Indirect Effect 3: -0.004, 95%CI [-0.02, 0.002]) with alcohol use (see Table S5, bottom). Standardized estimates are shown.

Right striatal associations. While we observed an indirect pathway linking left striatal activity to cannabis and e-cigarette use (main text Figure 3), similar effects were not detected for the right striatum. We offer a cautious interpretation. All study participants were right-handed and our MID implementation required a lateralized motor response (right index finger button press) which may have introduced hemispheric asymmetries in corticostriatal recruitment. While the association between handedness and brain laterization is complicated, striatal laterization “hotspots” have been identified in tasks requiring unilateral motor responses (34, 35). Thus, we view the left-striatal specificity as potentially reflecting action execution lateralization related to the task implementation rather than a substance-specific neurobiological signature of the left striatum per se.

**SUPPLEMENTAL REFERENCES**

1. Esteban O, Markiewicz CJ, Blair RW, Moodie CA, Isik AI, Erramuzpe A, et al. fMRIPrep: a robust preprocessing pipeline for functional MRI. Nature methods. 2019;16(1):111-6.

2. Gorgolewski K, Burns CD, Madison C, Clark D, Halchenko YO, Waskom ML, et al. Nipype: a flexible, lightweight and extensible neuroimaging data processing framework in python. Frontiers in neuroinformatics. 2011;5:13.

3. Tustison NJ, Avants BB, Cook PA, Zheng Y, Egan A, Yushkevich PA, et al. N4ITK: improved N3 bias correction. IEEE transactions on medical imaging. 2010;29(6):1310-20.

4. Avants BB, Epstein CL, Grossman M, Gee JC. Symmetric diffeomorphic image registration with cross-correlation: evaluating automated labeling of elderly and neurodegenerative brain. Medical image analysis. 2008;12(1):26-41.

5. Zhang Y, Brady M, Smith S. Segmentation of brain MR images through a hidden Markov random field model and the expectation-maximization algorithm. IEEE transactions on medical imaging. 2001;20(1):45-57.

6. Dale AM, Fischl B, Sereno MI. Cortical surface-based analysis: I. Segmentation and surface reconstruction. Neuroimage. 1999;9(2):179-94.

7. Klein A, Ghosh SS, Bao FS, Giard J, Häme Y, Stavsky E, et al. Mindboggling morphometry of human brains. PLoS computational biology. 2017;13(2):e1005350.

8. Fonov VS, Evans AC, McKinstry RC, Almli CR, Collins D. Unbiased nonlinear average age-appropriate brain templates from birth to adulthood. NeuroImage. 2009;47:S102.

9. Cox RW, Hyde JS. Software tools for analysis and visualization of fMRI data. NMR in Biomedicine: An International Journal Devoted to the Development and Application of Magnetic Resonance In Vivo. 1997;10(4‐5):171-8.

10. Greve DN, Fischl B. Accurate and robust brain image alignment using boundary-based registration. Neuroimage. 2009;48(1):63-72.

11. Jenkinson M, Bannister P, Brady M, Smith S. Improved optimization for the robust and accurate linear registration and motion correction of brain images. Neuroimage. 2002;17(2):825-41.

12. Power JD, Mitra A, Laumann TO, Snyder AZ, Schlaggar BL, Petersen SE. Methods to detect, characterize, and remove motion artifact in resting state fMRI. Neuroimage. 2014;84:320-41.

13. Behzadi Y, Restom K, Liau J, Liu TT. A component based noise correction method (CompCor) for BOLD and perfusion based fMRI. Neuroimage. 2007;37(1):90-101.

14. Satterthwaite TD, Elliott MA, Gerraty RT, Ruparel K, Loughead J, Calkins ME, et al. An improved framework for confound regression and filtering for control of motion artifact in the preprocessing of resting-state functional connectivity data. Neuroimage. 2013;64:240-56.

15. Lanczos C. Evaluation of noisy data. Journal of the Society for Industrial and Applied Mathematics, Series B: Numerical Analysis. 1964;1(1):76-85.

16. Miech RA, Johnston LD, Patrick ME, O’Malley PM. Monitoring the Future Study Annual Report. National survey results on drug use, 1975-2024: Overview and detailed results for secondary school students.: Institute for Social Research, University of Michigan; 2025.

17. Johnston LD, Miech RA, O’Malley, Bachman JGPM, Schulenberg JE, Patrick ME. Monitoring the Future national survey results on drug use 1975-2019: Overview, key findings on adolescent drug use. Institute for Social Research, University of Michigan; 2019.

18. Johnston LD, Miech RA, O’Malley, Bachman JGPM, Schulenberg JE, Patrick ME. Monitoring the Future national survey results on drug use 1975-2021: Overview, key findings on adolescent drug use. Institute for Social Research, University of Michigan; 2021.

19. Hoots BE, Li J, Hertz MF, Esser MB, Rico A, Zavala EY, et al. Alcohol and Other Substance Use Before and During the COVID-19 Pandemic Among High School Students - Youth Risk Behavior Survey, United States, 2021. MMWR Suppl. 2023;72(1):84-92.

20. Layman HM, Thorisdottir IE, Halldorsdottir T, Sigfusdottir ID, Allegrante JP, Kristjansson AL. Substance Use Among Youth During the COVID-19 Pandemic: a Systematic Review. Curr Psychiatry Rep. 2022;24(6):307-24.

21. Henneberger AK, Mushonga DR, Preston AM. Peer Influence and Adolescent Substance Use: A Systematic Review of Dynamic Social Network Research. Adolescent Research Review. 2021;6(1):57-73.

22. Kristjansson AL, Lilly CL, Thorisdottir IE, Allegrante JP, Mann MJ, Sigfusson J, et al. Testing risk and protective factor assumptions in the Icelandic model of adolescent substance use prevention. Health Educ Res. 2021;36(3):309-18.

23. Verdejo-Garcia A, Albein-Urios N. Impulsivity traits and neurocognitive mechanisms conferring vulnerability to substance use disorders. Neuropharmacology. 2021;183:108402.

24. Brumback T, Thompson W, Cummins K, Brown S, Tapert S. Psychosocial predictors of substance use in adolescents and young adults: Longitudinal risk and protective factors. Addict Behav. 2021;121:106985.

25. Okita K, Mandelkern MA, London ED. Cigarette Use and Striatal Dopamine D2/3 Receptors: Possible Role in the Link between Smoking and Nicotine Dependence. Int J Neuropsychopharmacol. 2016;19(11).

26. Jing C, An X, Fang J. A single-center case-control study of the association between dorsal striatal damage and nicotine addiction. Front Neurol. 2025;16:1553200.

27. Kim B, Kim HA, Woo J, Lee HJ, Kim TK, Min H, et al. Striatal Cholinergic Interneurons Control Physical Nicotine Withdrawal via Muscarinic Receptor Signaling. Adv Sci (Weinh). 2024;11(47):e2402274.

28. Kawamichi H, Sugawara SK, Hamano YH, Makita K, Kochiyama T, Sadato N. Increased frequency of social interaction is associated with enjoyment enhancement and reward system activation. Sci Rep. 2016;6:24561.

29. Trucco EM, Colder CR, Wieczorek WF. Vulnerability to peer influence: A moderated mediation study of early adolescent alcohol use initiation. Addictive behaviors. 2011;36(7):729-36.

30. Telzer EH, Jorgensen NA, Prinstein MJ, Lindquist KA. Neurobiological Sensitivity to Social Rewards and Punishments Moderates Link Between Peer Norms and Adolescent Risk Taking. Child Dev. 2021;92(2):731-45.

31. Crummy EA, O'Neal TJ, Baskin BM, Ferguson SM. One Is Not Enough: Understanding and Modeling Polysubstance Use. Front Neurosci. 2020;14:569.

32. Meyerhoff DJ. Structural Neuroimaging in Polysubstance Users. Curr Opin Behav Sci. 2017;13:13-8.

33. Yarkoni T, Poldrack RA, Nichols TE, Van Essen DC, Wager TD. Large-scale automated synthesis of human functional neuroimaging data. Nature methods. 2011;8(8):665-70.

34. Korponay C, Stein EA, Ross TJ. Laterality Hotspots in the Striatum. Cereb Cortex. 2022;32(14):2943-56.

35. Tejavibulya L, Horien C, Fredricks C, Ficek-Tani B, Westwater ML, Scheinost D. Brain handedness associations depend on how and when handedness is measured. Sci Rep. 2025;15(1):9674.
